# Genetic diversity and conservation in Bromeliaceae based on SSR markers

**DOI:** 10.1590/1678-4685-GMB-2023-0135

**Published:** 2024-04-26

**Authors:** Milene Ferreira Barcellos, Laís Mara Santana Costa, Fernanda Bered

**Affiliations:** 1 Universidade Federal do Rio Grande do Sul, Instituto de Biociências, Programa de Pós-Graduação em Genética e Biologia Molecular, Porto Alegre, RS, Brazil; 2 ConnectBio Soluções Biotecnológicas, Santa Cruz do Sul, RS, Brazil

**Keywords:** Bromeliads, Neotropics, population genetics, conservation genetics

## Abstract

Bromeliaceae has been used as a model Neotropical group to study evolutionary and diversification processes. Moreover, since large parts of the Neotropics are under anthropogenic pressure, a high percentage of possibly threatened species occurs. Despite this, concrete proposals for conservation based on genetic data are lacking. We compilated all genetic data obtained by nuclear microsatellites for Bromeliaceae and compared the levels of genetic diversity of subfamilies and their taxa, considering traits of life history and distribution in conservation and no conservation areas. We retrieved a total of 87 taxa (ca. 2.5% of the family size) and most present a mixed mating system, anemochoric dispersion, are ornithophilous, and were sampled outside Conservation Units, the majority occurring in the Atlantic Forest. Also, we found differences in some genetic indexes among taxa concerning seed dispersal mechanisms (e.g. Zoochoric taxa with higher diversity and lower inbreeding), mating systems (e.g. autogamous taxa showed higher inbreeding), outside/inside conservation units (allelic richness higher in not protected areas), and among different subfamilies (e.g. higher genetic diversity in Bromelioideae). The results obtained in this review can be useful for proposing conservation strategies, can facilitate the comparison of related taxa, and can help advance studies on the Bromeliaceae family.

## Introduction

The pineapple family (Bromeliaceae) has been consolidating as a good model to study evolutionary, biogeographical, and diversification processes. Diverse particularities make this family suitable for these studies, such as the wide range of morphological, physiological, and ecological traits, which are related to its complex biogeographical history (Givnish et al., 2011; Palma-Silva et al., 2016). Bromeliaceae is essentially endemic to the Americas and likely originated on the Guiana Shield (ca. 100 million years ago (mya)) and the diversification events within the family occurred in several stages, with the most significant radiation happening in the last 20 mya (Givnish *et al*., 2011). During this time, the family underwent rapid diversification, with the development of several subfamilies, genera, and species. Currently, the family harbors eight subfamilies (Brocchinioideae, Lindmanioideae, Tillandsioideae, Hechtioideae, Navioideae, Pitcairnioideae, Puyoideae and Bromelioideae) forming clades of different ages (Givnish *et al*., 2011). The diversification was likely facilitated by several key innovations, including epiphytism, the tank habit, leaf trichomes, CAM photosynthesis, and avian pollination (Givnish *et al*., 2014; Silvestro et al., 2014). 

Although Bromeliaceae is one of the richest groups in the Neotropics with more than 3,500 known species distributed from northern Patagonia to the southern USA (Benzing et al., 2000; Gouda *et al*., cont. updated), the geographic distribution of many species is poorly known, and this lack of knowledge can be extremely problematic in a conservation context since large parts of the Neotropics are under anthropogenic pressure (Antonelli, 2022). The continuous habitat loss is a cause of concern since many neotropical species are threatened and many of them may disappear even before being discovered or described by scientists (Lees and Pimm, 2015; Antonelli and Sanmartín 2011; Zizka et al., 2019). According to the IUCN Red List (http://www.iucnr edlist.org), only about 7% of Bromeliaceae were evaluated for threat criteria. Aiming to fill this gap, Zizka *et al*. (2019) compiled a strong dataset of bromeliads occurrence and modeled their geographic distribution. They confirm the Atlantic Forest, Andes, Central America, and southern Venezuela as centers of diversity and endemism of the family and concluded that about 81% of the evaluated species are probably threatened (highlighting the Atlantic Forest and Andean slopes). This scenario found for Bromeliaceae is not different from many other species, both animals and plants (Costello 2015; Boehm and Cronk, 2021). Despite this, the robustness and amount of genetic data used to propose conservation strategies can still be considered very incipient in several species (Andrello et al., 2022; Hoban et al., 2022). Incorporating genetic information into real-world conservation planning can still be considered a challenge despite the genetic composition (within-species genetic variation) being considered fundamental in maintaining biodiversity (Hoban *et al*., 2022; Shaw et al, 2023). The genetic composition is a measure of within-species diversity that helps in species adaptation and maintaining fitness (Hoban *et al*., 2022). The genetic data can be obtained directly through the analysis of genetic (e.g. DNA sequences and microsatellites) or genomic markers (e.g. RADseq family techniques and whole genome sequencing - WGS; Hohenlohe et al., 2021). Since the innovation of PCR and the subsequent use of nuclear microsatellite codominant markers for population genetics approaches, these markers have been successful and widely used. They provide the ability to screen many highly variable loci in virtually any species (Allendorf, 2017). The main metrics used to describe genetic differentiation, genetic diversity and inbreeding are *F*_ST_, *H*_*E*_ and *F*_*IS*_, respectively. Other important indexes are allelic richness and effective population size (Ottewell et al., 2016; Hoban *et al*., 2022). 

The population’s genetic diversity depends on species-specific life-history traits, past climatic and demographic events, and other population dynamics factors (Nybom, 2004; De Kort et al., 2021). In plants, factors such as the mating system, dispersal, and pollinating agents are essential in the distribution of genetic diversity. For example, wind-pollinated plant populations with high outcrossing rates and/or wind-dispersed seeds mostly maintain high genetic diversity due to the positive effects of these traits on effective population size (*N*_E_) and rates of molecular evolution (De Kort *et al*., 2021). For Bromeliaceae, despite the high percentage of possibly threatened species (Zizka et al., 2019), there are few concrete proposals for conservation based on genetic or genomic data (e.g. Lavor et al., 2014; Goetze et al., 2015; Cascante-Marín et al., 2020). Zanella et al. (2012) compiled all studies on Bromeliaceae from a genetic and evolutionary perspective, mainly in the context of population diversity. However, even though the number of studies has increased significantly since then, the few data on genetic diversity published until 2012 and reported in that article were not discussed taking into account the life history of the species, its distribution, and aspects such as seed dispersal mode and pollinators. These life history traits are expected to influence population genetic structure in plants and consequently are key factors for conservation recommendations and strategies in the current genomic era (Supple and Shapiro, 2018). To overview what the genetic data obtained so far for Bromeliaceae can tell us about the populations of different species, we analyzed some ecological aspects and their relationship with the main genetic indexes (e.g. *H*_*o*_, *H*_*E*_, *F*_*IS*_, *F*_*ST*_) obtained through microsatellite markers. Furthermore, we hypothesized that populations sampled within conservation units would present higher levels of genetic diversity when compared to those located outside protected environments. Therefore, we made this comparison based on the data collected. Here, we present the results of a systematic review of publications on patterns of intraspecific genetic diversity in the family. These data can be useful for managers and researchers in the design of conservation strategies that should be carried out individually for groups and species. 

## Material and Methods

### Data compilation

We searched published articles that used molecular markers for genetic diversity analysis in the Bromeliaceae family until April of 2023. We performed an extensive search for published sources comprising peer-reviewed articles, thesis and dissertations. Ten databases were used: Scholar Google, Brazilian Digital Library of Thesis and Dissertation, Wiley Online Library, Springer Link, SciELO, RefSeek, Microsoft Academic, Academic Search Premier, ResearchGate and Web of Science® (Institute of Scientific Information, Thomson Scientific). We considered different combinations of the keywords in three idioms (portuguese, English and Spanish): Bromeliaceae + [molecular markers], bromeliads + [molecular markers].

We retrieved studies that approached the intraspecific genetic diversity of different subfamilies of Bromeliaceae using the main molecular markers: simple sequence repeats (SSR), Restriction Fragment Length Polymorphism (RFLP), Random Amplified Polymorphic DNA (RAPD), Inter Simple Sequence Repeats (ISSR), Amplified Fragment Length Polymorphism (AFLP), Single Nucleotide Polymorphism (SNPs). We only considered studies that sampled non-cultivated populations. After the initial screening of studies, we decided to further analyze data on nuclear microsatellite markers due to three reasons: the amount of data already available from SSR on its own; the difficulty of making comparative analyses based on different markers; the interest that this marker has presented for decades in studies of population genetics and consequently in conservation genetics. Furthermore, of the 46 studies retrieved with other DNA markers (2 RFLP, 11 RAPD, 10 ISSR, 20 AFLP and 3 SNPs), the huge majority focused on approaches other than population genetics and worked with cultivated species, mainly of the genus *Ananas*.

For each study, we collected data on subfamily and species sampled, number of markers used, location, Conservation Status according to Official Lists and IUCN, whether any population was sampled inside a Conservation Unit, and genetic diversity parameters: allelic richness (AR), observed (*H*_*O*_) and expected (*H*_*E*_) heterozygosities, Inbreeding Coefficient (*F*_*IS*_) and Fixation Index (*F*_*ST*_). We also registered data on the life history traits of each species, whenever information was available: mating system, seed dispersal mechanisms, and pollinators. All of these ecological characteristics were obtained from the scientific articles retrieved for this review and were recorded according to the inference made by the authors. When the authors mentioned the exact species of pollinator, it was included within the following general categories: ornitophily, chiropterophily, and entomophily. When authors categorized the pollinator generically (e.g., vertebrates), this was retained. The geographical distribution of all populations of the sampled species considering each subfamily and their occurring biomes was registered when informed. In cases where the study didn’t present the geographic location of populations, we considered a general geographic point based on the description of the article.

The average of each genetic diversity parameter was calculated for each taxon considering all populations. When these data were not available for each population, we used those recorded for each SSR locus. We also registered which SSR markers were used in each subfamily, the total number of markers, and the transferability frequencies of each marker.

### Statistical analyses

We tested for differences in mating systems, pollinating agents, seed dispersal mechanisms, and subfamilies. In addition, we looked for differences between sampling inside or outside Conservation Units. Normal distribution (Shapiro-Wilk test) and equality of variances (Levene’s test) were verified for each parameter of genetic diversity (*H*_*O,*_ H_E_, *F*_*IS*_ and *F*_ST_) considering the groups of interest. Since most of the data did not meet the assumptions of the parametric tests, we opted for the use of non-parametric tests such as: Kruskal-Wallis test and Dunn’s post hoc followed by Benjamini-Hochberg false discovery rate (FDR) to correct the *P* values, and Mann-Whitney test. All analyses were performed in RStudio 2023.03.0.386 software (R Core Team, 2023), and the following packages were used: dplyr (Wickham *et al*., 2023), rstatix (Kassambara, 2021), RVAideMemoire (Hervé, 2021) and car (Fox and Weisberg, 2019).

## Results

We retrieved 75 studies using nuclear microsatellite markers in Bromeliaceae that met our pre-established criteria (Table S1). In these studies, a total of 87 taxa were contemplated, of which 82 belong to the three largest subfamilies: Bromelioideae (18 spp.), Pitcairnioideae (36 spp.), and Tillandsioideae (28 spp.) The other five species belong to the Puyoideae subfamily (Table S2). For Bromelioideae*, Aechmea* was the most sampled genus, with 10 species. For Pircairnioideae, the most frequently sampled genera were *Pitcairnia*, with 16 taxa, and *Dyckia* with eight taxa sampled. In the subfamily Tillandsioideae, *Vriesea* (ten taxa) and *Alcantarea* (eight taxa) were the most frequent genera (Table S2).

The genetic data obtained from 225 nuclear microsatellite markers were analyzed from 12,130 non-clonal individuals distributed in 722 populations. The average number of populations analyzed per study was 5.73, with a minimum of one population and a maximum of 65 populations. The number of individuals ranged from two to 526, with an average of 91.89 and the number of markers ranged from three to 19, with an average of 9.98. Allelic richness averaged 3.619, ranging from 1.145 to 9.510. The observed heterozygosity had a mean of 0.4213, with a minimum of 0.028 and a maximum of 1.000, while the expected heterozygosity ranged from 0.1410 to 0.8710, with a mean of 0.5109. *F*_*IS*_ values ranged from -0.1120 to 0.9170, with a mean of 0.2188, while *F*_*ST*_ averaged 0.2614, ranging from 0. 0128 to 0.8390 (Table 1).

**Table 1 -:** Parameters and indexes of genetic diversity obtained in this study, including the number of taxa, average, minimum and maximum for each variable. The number of populations and individuals analyzed in more than one study are repeated. RA: Allelic richness. *H*_*O*_: Observed heterozygosity. *H*_*E*_: Expected heterozygosity. *F*_*IS*_: Inbreeding coefficient. *F*_*ST*_: Fixation index.

Parameters	N Taxa^*^ (Total)	Average (min, max)
N populations	83 (722)	5.73 (1, 65)
N individuals	86 (12130)	91.89 (2, 526)
N markers	86 (225)	9.98 (3, 19)
Indexes	N Taxa	Average (min, max)
RA	63	3.619 (1.145, 9.510)
*H*_*O*_	86	0.4213 (0.028, 1.000)
*H*_*E*_	86	0.5109 (0.1410, 0.8710)
*F*_*IS*_	30	0.2188 (-0.1120, 0.9170)
*F*_*ST*_	30	0.2614 (0. 0128, 0.8390)

^*^The number of taxa includes those taxa that are repeated (analyzed in more than one article).

The number of nuclear microsatellite loci developed in the studies evaluated was 42 markers for Bromelioideae, 102 for Pitcairnioideae, 57 for Tillandsioideae, and 24 for Puyoideae totaling 225 microsatellite loci. The markers originally developed for species of a certain subfamily that were used in species of other subfamilies, as well as the frequency of transferability of these markers can be seen in Figure S1. 

**Figure 1 -:**
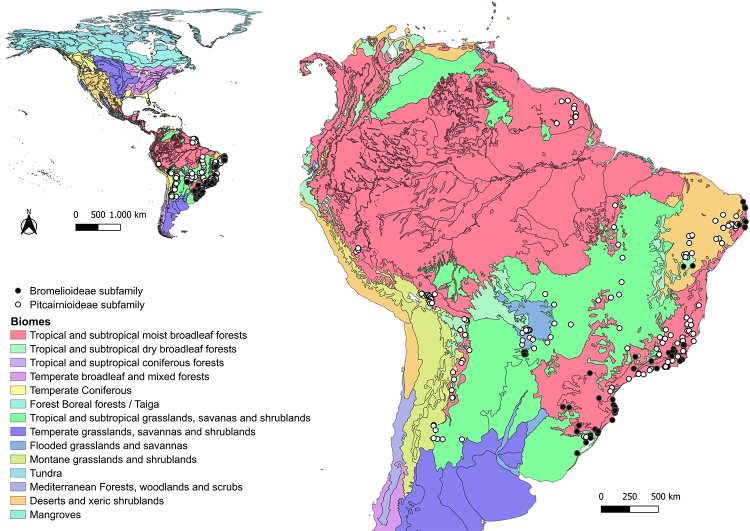
Geographic distribution of taxa belonging to the Bromelioideae and Pitcairnioideae subfamilies retrieved using nuclear microsatellite markers from literature. Biomes according to the World Wildlife Fund (WWF).

**Figure 2 -:**
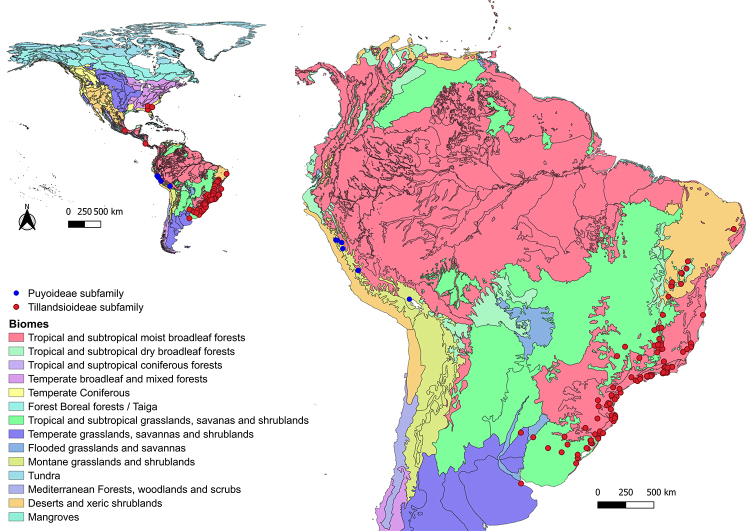
Geographic distribution of taxa belonging to the Puyoideae and Tillandsioideae subfamilies retrieved using nuclear microsatellite markers from literature. Biomes according to the World Wildlife Fund (WWF).

Of all the studies retrieved, most analyzed species have a mixed mating system, anemochoric dispersion, ornithophilous syndrome (Table S3), and were sampled outside Conservation Units (data not shown), most occurring in the tropical and subtropical moist broadleaf forests biome (Atlantic Forest; Table S3; Figures 1 and 2). The analyzed populations of the Bromelioideae subfamily were concentrated in the tropical and subtropical moist broadleaf forests biome (Atlantic Forest and Pampa) with samples in the Deserts and xeric shrublands biome (Caatinga) and flooded grasslands and savannas biome (Pantanal; Table S3; Figure 1). Populations of Pitcairnoidae were more frequently sampled in the Caatinga and Atlantic Forest biomes, in addition to populations sampled in French Guiana, Bolivia and Argentina (Table S3; Figure 1). The subfamily Tillandsioideae had the highest frequency of populations sampled in the Atlantic Forest and Pampa, also with populations sampled in the Caatinga and Cerrado, as well as in Mexico and Panama. The Puyoideae sampled are restricted to the Andine region (Montane grasslands and shrublands biome; Table S3; Figure 2).

Genetic diversity in Bromeliaceae

**Figure 3 -:**
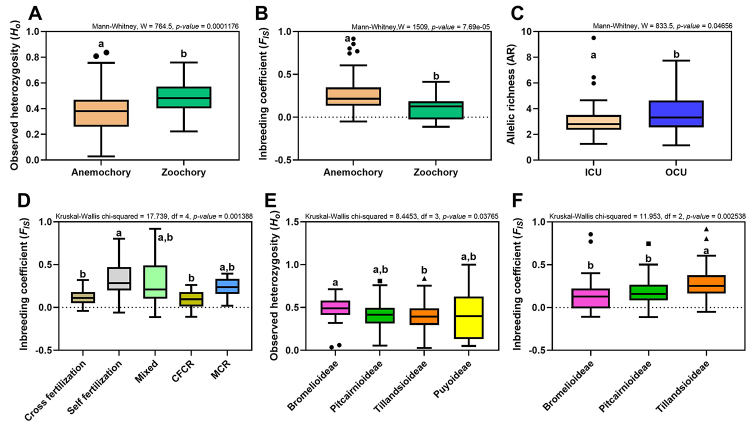
Box and whisker plots showing the statistically significant comparisons in this study. a) *H*o and b) *F*_*IS*_ for seed dispersal mechanisms, respectively. c) Allelic Richness (AR) for the comparisons of taxa sampled inside and outside the conservation units. d) *F*_*IS*_ for the reproductive system. e) *H*o and f) *F*_*IS*_ for subfamily comparisons, respectively. Boxes show the 25th, 50th and 75th percentiles; whiskers depict the minimum and maximum values of each variable. *P* ≤ 0.05. Mixed: mixed mating system; ICU: inside conservation unit; OCU: outside conservation unit; CFCR: Cross fertilization and clonal reproduction; MCR: Mixed and clonal reproduction

Some genetic indexes analyzed were significantly different considering the ecological aspects of the sampled species. Our results pointed out differences between taxa with different seed dispersal mechanisms. Zoochoric taxa revealed higher *H*_*O*_ (Figure 3A) and lower *F*_*IS*_ (Figure 3B) when compared with anemochoric ones. When comparing taxa sampled inside conservation units to those sampled outside, we found that the allelic richness was higher in plants collected in not protected areas (Figure 3C). Concerning the mating systems, we found that the *F*_*IS*_ of autogamous taxa is higher than those that present cross-fertilization and cross-fertilization and clonal reproduction (CFCR; Figure 3D). The *H*_*O*_ and *F*_*IS*_ were also different when comparing the subfamilies. Bromelioideae presented higher *H*_*O*_ and lower *F*_*IS*_ when compared with Tillandsioideae (Figures 3E and 3F respectively). We did not find differences among species with different pollinating agents concerning all indexes tested (Figure S2). Other parameters that did not present differences considering some genetic indices are presented in Figures S3 and S4.

Regarding species conservation status, the studies retrieved revealed that most taxa are not listed in any threat category (NOL), both in national and subnational lists (Figure S5). In the National lists (Figure S5A), Pitcairnoideae was the subfamily with more species categorized as Data Deficient (DD), while representatives of the subfamily Bromelioideae were categorized more frequently in the Vulnerable (VU) category. The Tillandsioideae subfamily was more representative in the Least Concern category (LC). At the sub-national level (Figure S5B), the Tillandsioideae and Pitcairnoideae subfamilies had their highest frequencies in the Vulnerable (VU) category, while the Bromelioideae subfamily was equally frequent in the Vulnerable (VU) and Endangered (EN) categories. 

## Discussion

The distribution and magnitude of genetic diversity in plant populations are related to ecological factors and species life history traits (Loveless and Hamrick, 1984; Nybom, 2004). In Bromeliaceae, although various genetic data have already been accumulated, mainly using microsatellite markers, they were not compiled to obtain insights or even propose more consistent species conservation strategies to the family as a whole. The genetics and genomics concepts, together with the ability to efficiently study genetic factors, are well-established as important factors for quantifying and mitigating threats to natural populations (Hohenlohe et al., 2021). Although many conservation researchers are in the process of transferring from genetics to genomics, several studies indicate that the data obtained by microsatellites are informative to answer numerous questions (e.g. those related to diversity within populations, differentiation between populations, and inbreeding), in addition to being much less expensive and therefore more accessible for a greater number of researchers (Puckett, 2017; Vashistha et al., 2020).

In this review, we retrieved 87 Bromeliaceae taxa studied using microsatellite markers (Table S2). According to Gouda *et al*. (cont. updated), there are 3590 species of bromeliads distributed in 75 genera. Considering the most frequently studied genera in the works recovered in this review (Table S2), of the 244 species of the genus *Aechmea*, ten (4.1%) were studied; of the 411 species of the genus *Pitcairnia*, 16 (3.9%) were evaluated; of the 179 of the genus *Dyckia*, eight (4.4%) were studied, of the 214 species of the genus *Vriesea*, only ten (4.6%) were evaluated; and of the 47 of the genus *Alcantarea*, eight (17%) were studied. This demonstrates that most species and genera of the Bromeliaceae family are still not included in studies of genetic diversity, even so with nuclear microsatellites, the currently most used markers for this approach. Even the most numerous genera (*Aechmea*, *Dyckia*, *Pitcairnia, Vriesea* and *Tillandsia*) have a proportionally low frequency in these studies. These results highlight the need for further genetic studies with species of the Bromeliaceae family, using microsatellites or any other genomic approach.

Of the ecological aspects evaluated, only the pollinating agents showed no difference concerning the genetic indexes evaluated (Figure S2). Mobile pollinators tend to conduct greater gene flow within and among populations, compared to less mobile pollinators, and these differences may influence patterns of population structure in different species (Wessinger, 2021). However, we could not capture differences in the diversity indices and genetic distribution related to the pollinating agents of the species retrieved here (Figure S2). Gamba and Muchhala (2023) highlighted that pollen dispersal depends on the foraging behavior of pollinators and this capacity can vary significantly between animals from the same taxonomic group, such as insects. In their study, they found that plants pollinated by large insects, such as euglossine bees, can connect plant populations as effectively as some kinds of hummingbirds. Here, we classify the species’ pollinating agent generically (entomophily, for example) due to the often incomplete information reported in the retrieved articles. A more detailed classification of pollinators could have yielded different results in our analyses.

Considering the seed dispersal factor, we found that species considered zoochoric have a higher *H*o and a lower *F*_*IS*_ (Figure 3). In fact, several authors have discussed that seed dispersal mediated by animals should prevent population divergence, reduce inbreeding, and increase the levels of genetic diversity (Hamrick et al., 1993; Nazareno et al., 2021). On the other hand, wind-dispersed species (especially the epiphytes), tend to disperse closer to the mother plant and consequently may increase inbreeding (Hamrick *et al*., 1993; Bullock and Clarke, 2000). Paggi et al. (2010) revealed that the seeds of the epiphyte and anemochoric bromeliad *Vriesea gigantea* Gaud. dispersed over short distances because, besides the seeds becoming trapped in the forest matrix, those within the same capsule tend to become entangled with the plumose appendages and therefore immobilized. Regarding conservation propositions, species capable of long-distance dispersion, such as the zoochoric, conservation strategies should focus on protecting large populations to encompass the entire genetic diversity of the species (Swift et al., 2016). In contrast, species with seeds wind-dispersed tend to have most of the genetic diversity distributed among different populations; in this case, as each population has a unique set of alleles, it is necessary to conserve a larger number of small populations to protect the genetic diversity (Ottewell et al., 2016; Swift *et al*., 2016; Allendorf, 2017).

Flowering plants exhibit a remarkable diversity of mating systems, ranging from obligate outcrossing, and apomixis to nearly complete selfing, and the mating system can have a profound impact on the genetic diversity of populations (Loveless and Hamrick, 1984; Yi et al., 2022). Analyzing the different bromeliad mating systems, we found that outcrosser species show lower *F*_*IS*_ when compared to the selfing species (Figure 3). In fact, outcrossing generally reduces population subdivision, increases heterozygosities and decreases inbreeding, while selfing tends to increase the potential for genetic differentiation among populations, decreases within-population genetic diversity, and increases inbreeding (Loveless and Hamrick, 1984). Cascante-Marín et al. (2020) studying populations of the dioecious bromeliad *Aechmea mariae reginae* found high genetic diversity as expected for obligate outcrossing plants. However, some populations showed significant inbreeding, probably due to biparental inbreeding. The authors highlighted that although some recent gene flow was detected among the clusters found, the studied populations showed high genetic differentiation and further reduction and fragmentation of their natural habitat is likely to increase genetic isolation and the long-term viability of this species can be threatened. Thus, from a conservation standpoint, understanding how the mating system affects the variation in the genetic diversity within and between populations of a species in particular, is essential for its conservation. Conservation actions must be planned taking into account the different modes of the mating system: for selfing species, it is necessary to protect many populations to fully encompass genetic diversity since these species tend to present most of their genetic diversity among populations and not within populations. As for outcrossing, which maintains most of the genetic diversity within populations, conservation actions should focus on protecting a few large populations. This probably will be sufficient to fully contemplate the genetic diversity of a species (Ottewell et al., 2016; Swift et al., 2016).

Contrary to our hypothesis, the allelic richness of the sampled species was significantly higher in non-protected areas, outside conservation units (Figure 3). Protected areas should ideally promote the long-term persistence of populations, maintaining the neutral evolutionary processes that regulate genetic diversity and enable species and populations to overcome changes in their surrounding environments (Hanson et al., 2020). In a wide range of taxa, habitat fragmentation has been commonly related to a reduction in genetic diversity (DiBattista, 2008). However, in addition to some genetic-variation metrics being more sensitive than others to demographic changes and habitat disturbance, plant genetic patterns can be related to several aspects of their biology, such as their life form, mating system, or commonness (González *et al*., 2020). Furthermore, our data set represents a wide variety of biomes (Table S3; Figures 1 and 2) and habitat heterogeneity might impose differential selection pressure on populations across the landscape, influencing genetic diversity (Ballesteros-Mejia et al., 2020). Other factors that must be taken into account to explain this result are: the time taken the sampled area has been protected; the original genetic diversity before the area was protected and the type of molecular marker used for the analysis. Waqar et al. (2021), studying the genetic diversity of a tropical tree in protected areas located in Atlantic Forest fragments, observed a substantial decay in genetic variability between generations, indicating the need to implement measures to improve habitat protection and expand forest restoration projects. Ballesteros-Mejia *et al*. (2020) investigated the effectiveness of the Cerrado conservation units compiling genetic information obtained from different types of molecular markers. They found different results when comparing sequences with low evolutionary rates (e.g. chloroplast DNA) and microsatellite loci. The authors did not detect a loss of genetic diversity caused by fragmentation using microsatellites and attributed this result to the high evolutionary rates of this marker that increase heterozygosity and hinder the fragmentation effects. 

Aiming to infer the amount of genetic diversity and how it is distributed in populations, we evaluated the differences in the genetic parameters evaluated among the Bromeliaceae subfamilies. Bromelioideae, represented by 18 taxa in this review, presented higher *H*o (reflecting greater genetic diversity) than subfamily Tillandsioideae, represented by 28 taxa. On the other hand, Tillansioideae showed higher *F*_*IS*_ than Bromelioideae and Pitcairnoideae (Figure 3). The subfamily Bromelioideae harbors 986 species (Gouda *et al*., cont. updated) and is a highly diverse group, being the Atlantic rainforest its center of diversity and endemism (Givnish et al., 2011; Goetze et al., 2016). Tillandsioideae is the largest (c. 1500 species) and the most widespread subfamily of Bromeliaceae, being Central America an important radiation Center (Givnish *et al*., 2011). Zizka et al (2019) compiled a dataset of occurrence records of more than 90% of Bromeliaceae species and modeled their geographic distribution combined with information on taxonomy to infer the conservation status of the taxa. The results revealed that 73% of Tillansioideae taxa are threatened and Bromelioideae has 82% of its taxa possibly threatened. These results do not take into account genetic data and contrast with the results obtained here. Although the sample of genetic data from these two subfamilies is much smaller in our review, the results point to greater genetic diversity and less inbreeding in Bromelioideae populations (e.g. *Bromelia hieronymi* Mez, *Sincoraea ophiuroides* (Louzada & Wand.), *Aechmea comata* (Gaudich.) Baker, Table S3), which would potentially minimize the degree of threat. Although we are aware of the limitations of comparing results with such different approaches and magnitudes, it is evident that genetic data must be included in propositions of strategies for species conservation. For these strategies, our intention here is not to design a single plan for such a highly diverse family. Instead, we propose that managers and researchers use the data compiled here to assist in the design of strategies that will be carried out individually for groups and species. 

In summary, considering that Bromeliaceae is a model to understand the evolutionary history of the Neotropics (Silvestro et al., 2014; Zizka et al., 2019) and that the lack of knowledge of the geographic distribution and genetic diversity of many species and populations may be especially challenging in the context of advancing human pressure in the Neotropics (Zizka *et al*., 2019), we think that the compilation made in this review can be useful in different ways. First, to map, within the geographic distribution of the family, which are the best-represented biomes and taxa concerning potentially useful genetic data for proposing conservation strategies. Second, the compilation of results from genetic data, using the indices most used by population geneticists, will facilitate comparison with new studies on related taxa. Finally, the gaps found in this review will serve to boost new and necessary studies on the Bromeliaceae family. 

## Supplementary material

The following online material is available for this article:

Table S1 - Total of sampled taxa for each subfamily in the 75 studies with microsatellites analyzed in this article.

Table S2 - Subfamilies, taxa, number of populations (N pops), sampled individuals (N ind), nuclear microsatellite markers used (N SSRs), average of genetic diversity parameters (Allelic Richness - AR, *H*o, *H*_*E*_ , *F*_*IS*_, *F*_*ST*_), Mating System, Seed Dispersal Mechanism, Pollination Syndrome, Biome, National Conservation (NC) and Local Conservation (LC) Status for each taxa analyzed in the 75 studies considered in the present work. Acronyms: BA (Bahia); ES (Espírito Santo); MG (Minas Gerais); MS (Mato Grosso do Sul); PB (Paraíba); PE (Pernambuco); PR (Paraná); RJ (Rio de Janeiro); RN (Rio Grande do Norte); RS (Rio Grande do Sul); SC (Santa Catarina); SE (Sergipe); SP (São Paulo); AR (Argentina); BO (Bolivia); CO (Colombia); CR (Costa Rica); EC (Ecuator); GF (French Guiana); MX (Mexico); PE (Peru); US (United States). NA: Not Threatened; DD: Data Deficient; LC: Least Concern; NT: Near Threatened; VU: Vulnerable; EN: Endangered; CR: Critically Endangered. - no data available.

Table S3 - All 75 studies analyzed for the genetic diversity of the Bromeliaceae family based on SSR markers.

Figure S1 - Microsatellite markers originally developed for species of subfamilies Bromelioideae, Pitcairnoideae and Tillandsioideae used in species of other subfamilies and the frequency of transferability of these markers.

Figure S2 - Box and whisker plots showing the comparisons that were not statistically significant in this study considering a *p* ≤ 0.05.

Figure S3 - Box and whisker plots showing the comparisons that were not statistically significant in this study considering a *P* ≤ 0.05.

Figure S4 - Box and whisker plots showing the comparisons that were not statistically significant in this study considering a *p* ≤ 0.05.

Figure S5 - Conservation status of the taxa distributed in the four subfamilies retrieved in this study and their threat category.

## References

[B1] Allendorf FW (2017). Genetics and the conservation of natural populations: Allozymes to genomes. Mol Ecol.

[B2] Andrello M, D’Aloia C, Dalongeville A, Escalante MA, Guerrero J, Perrier C, Torres-Florez JP, Xuereb A, Manel S (2022). Evolving spatial conservation prioritization with intraspecific genetic data. Trends Ecol Evol.

[B3] Antonelli A, Sanmartín I (2011). Why are there so many plant species in the Neotropics?. Taxon.

[B4] Antonelli A (2022). The rise and fall of Neotropical biodiversity. Bot J Linn Soc.

[B5] Ballesteros-Mejia L, Lima JS, Collevatti RG (2020). Spatially-explicit analyses reveal the distribution of genetic diversity and plant conservation status in Cerrado biome. Biodivers Conserv.

[B6] Benzing DH, Bennett B, Brown G, Dimmitt M, Luther H, Ramirez I, Terry R, Till W (2000). Bromeliaceae. Profile of an adaptive radiation.

[B7] Boehm MMA, Cronk QCB (2021). Dark extinction: The problem of unknown historical extinctions. Biol Lett.

[B8] Bullock JM, Clarke RT (2000). Long distance seed dispersal by wind: Measuring and modelling the tail of the curve. Oecologia.

[B9] Cascante-Marín A, Trejos C, Madrigal R, Fuchs EJ (2020). Genetic diversity and reproductive biology of the dioecious and epiphytic bromeliad Aechmea mariae-reginae (Bromeliaceae) in Costa Rica: Implications for its conservation. Bot J Linn Soc.

[B10] Costello MJ (2015). Biodiversity: The known, unknown, and rates of extinction. Curr Biol.

[B11] De Kort H, Prunier JG, Ducatez S, Honnay O, Baguette M, Stevens VM, Blanchet S (2021). Life history, climate and biogeography interactively affect worldwide genetic diversity of plant and animal populations. Nat Commun.

[B12] DiBattista JD (2008). Patterns of genetic variation in anthropogenically impacted populations. Conserv Genet.

[B13] Fox J, Weisberg S (2019). An R Companion to Applied Regression.

[B14] Gamba D, Muchhala N (2023). Pollinator type strongly impacts gene flow within and among plant populations for six Neotropical species. Ecology.

[B15] Givnish TJ, Barfuss MHJ, Van Ee B, Riina R, Schulte K, Horres R, Gonsiska PA, Jabaily RS, Crayn DM, Smith JAC (2014). Adaptive radiation, correlated and contingent evolution, and net species diversification in Bromeliaceae. Mol Phylogenet Evol.

[B16] Givnish TJ, Barfuss MHJ, van Ee B, Riina R, Schulte K, Horres R, Gonsiska PA, Jabaily RS, Crayn DM, Smith JAC (2011). Phylogeny, adaptive radiation, and historical biogeography in Bromeliaceae: Insights from an eight-locus plastid phylogeny. Am J Bot.

[B17] Goetze M, Büttow MV, Zanella CM, Paggi GM, Bruxel M, Pinheiro FG, Sampaio JAT, Palma-Silva C, Cidade FW, Bered F (2015). Genetic variation in Aechmea winkleri, a bromeliad from an inland Atlantic rainforest fragment in Southern Brazil. Biochem Syst Ecol.

[B18] Goetze M, Schulte K, Palma-Silva C, Zanella CM, Büttow M V., Capra F, Bered F (2016). Diversification of Bromelioideae (Bromeliaceae) in the Brazilian Atlantic rainforest: A case study in Aechmea subgenus Ortgiesia. Mol Phylogenet Evol.

[B19] González A V., Gómez-Silva V, Ramírez MJ, Fontúrbel FE (2020). Meta-analysis of the differential effects of habitat fragmentation and degradation on plant genetic diversity. Conserv Biol.

[B20] Hamrick JL, Murawski DA, Nason JD (1993). The influence of seed dispersal mechanisms on the genetic structure of tropical tree populations. Vegetatio.

[B21] Hanson JO, Marques A, Veríssimo A, Camacho-Sanchez M, Velo-Antón G, Martínez-Solano Í, Carvalho SB (2020). Conservation planning for adaptive and neutral evolutionary processes. J Appl Ecol.

[B22] Hoban S, Archer FI, Bertola LD, Bragg JG, Breed MF, Bruford MW, Coleman MA, Ekblom R, Funk WC, Grueber CE (2022). Global genetic diversity status and trends: Towards a suite of Essential Biodiversity Variables (EBVs) for genetic composition. Biol Rev.

[B23] Hohenlohe PA, Funk WC, Rajora OP (2021). Population genomics for wildlife conservation and management. Mol Ecol.

[B24] Lavor P, van den Berg C, Jacobi CM, Carmo FF, Versieux LM (2014). Population genetics of the endemic and endangered Vriesea minarum (Bromeliaceae) in the Iron Quadrangle, Espinhaço Range, Brazil. Am J Bot.

[B25] Lees AC, Pimm SL (2015). Species, extinct before we know them?. Curr Biol.

[B26] Loveless M, Hamrick J (1984). Ecological determinants. Annu Rev Ecol Syst.

[B27] Nazareno AG, Knowles LL, Dick CW, Lohmann LG (2021). By animal, water, or wind: Can dispersal mode predict genetic connectivity in riverine plant species?. Front Plant Sci.

[B28] Nybom H (2004). Comparison of different nuclear DNA markers for estimating intraspecific genetic diversity in plants. Mol Ecol.

[B29] Ottewell KM, Bickerton DC, Byrne M, Lowe AJ (2016). Bridging the gap: A genetic assessment framework for population-level threatened plant conservation prioritization and decision-making. Divers Distrib.

[B30] Paggi GM, Sampaio JAT, Bruxel M, Zanella CM, Goetze M, Büttow MV, Palma-Silva C, Bered F (2010). Seed dispersal and population structure in Vriesea gigantea, a bromeliad from the Brazilian. Bot J Linn Soc.

[B31] Palma-Silva C, Leal BSS, Chaves CJN, Fay MF (2016). Advances in and perspectives on evolution in Bromeliaceae. Bot J Linn Soc.

[B32] Puckett EE (2017). Variability in total project and per sample genotyping costs under varying study designs including with microsatellites or SNPs to answer conservation genetic questions. Conserv Genet Resour.

[B33] Shaw RE, Spencer PB, Gibson LA, Dunlop JA, Kinloch JE, Mokany K, Byrne M, Moritz C, Davie H, Travouillon KJ (2023). Linking life history to landscape for threatened species conservation in a multiuse region. Conserv Biol.

[B34] Silvestro D, Zizka G, Schulte K (2014). Disentangling the effects of key innovations on the diversification of bromelioideae (Bromeliaceae). Evolution.

[B35] Supple MA, Shapiro B (2018). Conservation of biodiversity in the genomics era. Genome Biol.

[B36] Swift JF, Smith SA, Menges ES, Bassüner B, Edwards CE (2016). Analysis of mating system and genetic structure in the endangered, amphicarpic plant, Lewton’s polygala (Polygala lewtonii). Conserv Genet.

[B37] Vashistha G, Deepika S, Dhakate PM, Khudsar FA, Kothamasi D (2020). The effectiveness of microsatellite DNA as a genetic tool in crocodilian conservation. Conserv Genet Resour.

[B38] Waqar Z, Moraes RCS, Benchimol M, Morante-Filho JC, Mariano-Neto E, Gaiotto FA (2021). Gene flow and genetic structure reveal reduced diversity between generations of a tropical tree, Manilkara multifida Penn., in Atlantic Forest fragments. Genes (Basel).

[B39] Wessinger CA (2021). From pollen dispersal to plant diversification: Genetic consequences of pollination mode. New Phytol.

[B40] Yi H, Wang J, Wang J, Rausher M, Kang M (2022). Genomic insights into inter- and intraspecific mating system shifts in Primulina. Mol Ecol.

[B41] Zanella CM, Janke A, Palma-Silva C, Kaltchuk-Santos E, Pinheiro FG, Paggi GM, Soares LES, Goetze M, Büttow MV, Bered F (2012). Genetics, evolution and conservation of Bromeliaceae. Genet Mol Biol.

[B42] Zizka A, Azevedo J, Leme E, Neves B, da Costa AF, Caceres D, Zizka G (2019). Biogeography and conservation status of the pineapple family (Bromeliaceae). Divers Distrib.

