# Supplementary material for: Genetic diversity and conservation in Bromeliaceae based on SSR markers
Source: Genet Mol Biol. 2024 Apr 26;46(3 Suppl 1):e20230135. doi: 10.1590/1678-4685-GMB-2023-0135 (PMC11113272; doi:10.1590/1678-4685-GMB-2023-0135)
Supplement: Table S1 - [file 1415-4757-GMB-46-03-s1-e20230135-s1.pdf]

*Genetics and Molecular Biology*

https://doi.org/10.1590/1678-4685-GMB-2023-0135

# Supplementary Material to "Genetic diversity and conservation in Bromeliaceae based on SSR markers"

**Table S1 -** All 75 studies analyzed for the genetic diversity of the Bromeliaceae family based on SSR markers.

| Number | Title | Authors | Year | Journal (vol, pages) |
|---|---|---|---|---|
| 1 | *Polymorphic microsatellites for the study of fragmented populations of Pitcairnia geyskesii L. B. Smith (Bromeliaceae), a specific saxicolous species of inselbergs in French Guiana* | Sarthou, C.; Boisselier-Dubayle, M.C.; Samadi, S. | 2003 | Molecular Ecology Notes 3, 221–223 |
| 2 | *Blackwell Publishing Ltd Population differentiation and species cohesion in two closely related plants adapted to neotropical high-altitude 'inselbergs', Alcantarea imperialis and Alcantarea geniculate (Bromeliaceae)* | Barabará, T.; Martinelli, G.; Fay, F.; Mayo, S.J. and Lexer, C. | 2007 | Molecular Ecology 16, 1981–1992 |
| 3 | *A set of polymorphic microsatellite loci for Vriesea gigantea and Alcantarea imperialis (Bromeliaceae) and cross-amplification in other bromeliad species* | Palma-Silva, C.; Cavallari, M.M.; Barbará, T.; Lexer, C.; Gimenes, M.A.; Bered, F. and Bodanese-Zanetti, M.H. | 2007 | Molecular Ecology Notes 7, 654–657 |
| 4 | *Within-population spatial genetic structure in four naturally fragmented species of a neotropical inselberg radiation, Alcantarea imperialis, A. geniculata, A. glaziouana and A. regina (Bromeliaceae)* | Barbará, T.; Lexer, C.; Martinelli, G.; Mayo, S.J.; Fay, F. and Heuertz, M. | 2008 | Heredity 101, 285–296 |
| 5 | *Isolation and haracterization of microsatellite loci in Pitcairnia albiflos (Bromeliaceae), an endemic bromeliad from the Atlantic Rainforest, and cross-amplification in other species* | Paggi, G.M.; Palma-Silva, C.; Bered, F.; Cidade, F.W.; Sousa, A.C.B.; Souza, A.P.; Wendt, T. and Lexer, C. | 2008 | Molecular Ecology Resources 8, 980–982 |
| 6 | *Genetic relationships and variation in reproductive strategies in four closely* | Barabará, T.; Martinelli, G.; Palma- | 2009 | Annals of Botany 103: 65–77 |

| Number | Title | Authors | Year | Journal (vol, pages) |
|---|---|---|---|---|
| | *related bromeliads adapted to neotropical 'inselbergs': Alcantarea glaziouana, A. regina, A. geniculata and A. imperialis (Bromeliaceae)* | Silva, C.; Fay, F.; Mayo, S.J. and Lexer, C. | | |
| 7 | *Range-wide patterns of nuclear and chloroplast DNA diversity in Vriesea gigantea (Bromeliaceae), a neotropical forest species* | Palma-Silva, C.; Lexer, C.; Paggi, G.M.; Barabará, T.; Bered, F. and Bodanese-Zanetti, M.H. | 2009 | Heredity 103, 503–512 |
| 8 | *Genetic structure of the xerophilous bromeliad Pitcairnia geyskesii on inselbergs in French Guiana a test of the forest refuge hypothesis* | Boisselier-Dubayle, M.C.; Leblois, R.; Samadi, S.; Lambourdière, J. and Sarthou, C. | 2010 | Ecography, 33: 175-184 |
| 9 | *Low Genetic Diversity in Tillandsia recurvata (Bromeliaceae), the Most Ubiquitous Epiphyte Species of the Semiarid and Arid Zones of North America* | Solórzano, S.; Solís, S.J. and Dávila, P. | 2010 | Journal of the Bromeliad Society 60(2), 71-81 |
| 10 | *Sympatric bromeliad species (Pitcairnia spp.) facilitate tests of mechanisms involved in species cohesion and reproductive isolation in Neotropical inselbergs* | Palma-Silva, C.; Wendt, T.; Pinheiro, F.; Barbará, T. and Fay, M.F. | 2011 | Molecular Ecology 20, 3185–3201 |
| 11 | *Genetic Structure and Phenotypic variation in wild populations of the medicinal tetraploid species Bromelia antiacantha (Bromeliaceae)* | Zanella, C.M.; Bruxel, M.; Paggi, G.M.; Goetze, M.; Buttow, M.V.; Cidade, F.W. and Bered, F. | 2011 | American Journal of Botany 98(9): 1511–1519 |
| 12 | *Transferability and characterization of microsatellite markers in five Bromeliaceae species belonging to the subfamilies Pitcairnoideae and Bromelioideae* | Miranda, F.D.; Gontijo, A.B.P.L.; Santiliano, F.C.; Favoreto, F.C. and Soares, T.C.B. | 2012 | Biota Neotropica 12 (3), 319-323 |
| 13 | *Development of Microsatellite markers in Fosterella rusbyi (Bromeliaceae) using 454 Pyrosequencing* | Wohrmann, T.; Wagner, N.; Krapp, F.; Huettel, B. and Weising, K. | 2012 | American Journal of Botany: e160–e163 |
| 14 | *Microsatellites in the Endangered Species Dyckia distachya (Bromeliaceae) and Cross-Amplification in Other Bromeliads* | Zanella, C.M.; Janke, A.; Paggi, G.M.; Goetze, M.; Reis, M.S. and Bered, F. | 2012 | Int. J. Mol. Sci. 13, 15859-15866 |

| Number | Title | Authors | Year | Journal (vol, pages) |
|---|---|---|---|---|
| 15 | *Development of microsatellite markers for genetic diversity analysis of Aechmea caudata (Bromeliaceae) and cross-species amplification in other bromeliads* | Goetze, M.; Louzada, R.B.; Wanderlay, M.G.L.; Souza, L.M.; Bered, F. and Palma-Silva, C. | 2013 | Biochemical Systematics and Ecology 48, 194–198 |
| 16 | *Development of SSR Markers for Encholirium horridum (Bromeliaceae) and Transferability to Other Pitcairnioideae* | Hmeljevski, K.V.; Ciampi, M.B.; Balduf, C.; Reis, M.S. and Forzza, R.C. | 2013 | Applications in Plant Sciences, 1 (4): 1200445 |
| 17 | *Transferability of 10 nuclear microsatellite primers to Vriesea minarum (Bromeliaceae), a narrowly endemic and threatened species from Brazil* | Lavor, P; Van der Berg, C. and Versieux, L.M. | 2013 | Brazilian Journal of Botany 36, 165–168 |
| 18 | *Development of 15 nuclear microsatellite markers in the genus Dyckia (Pitcairnioideae; Bromeliaceae) using 454 pyrosequencing* | Wohrmann, T.; Pinangé, D.S.B.; Krapp, F.; Benko-Iseppon, A.M.; Huettel, B. and Weising, K. | 2013 | Conservation Genet Resour 5:81–84 |
| 19 | *Microsatellite Loci for Orthophytum ophiuroides (Bromelioideae, Bromeliaceae) Species Adapted to Neotropical Rock Outcrops* | Aoki-Gonçalvez, F.; Louzada, R.B.; Souza, L.M. and Palma-Silva, C. | 2014 | Applications in Plant Sciences 2 (3): 1300073 |
| 20 | *Genetic Diversity and Spatial Genetic Structure of an Epiphytic Bromeliad in Costa Rican Montane Secondary Forest Patches* | Cascante-Marín, A.; Oostermeijer, G.; Wolf, J. and Fuchs, E.J. | 2014 | Biotropica 46 (4), 425-432 |
| 21 | *Population genetics of the endangered Vriesea minarum (Bromeliaceae) in the Iron Quadrangle, Espinhaço Range, Brazil* | Lavor, P; Van der Berg, C.; Jacobi, C.M.; Carmo, F.F. and Versieux, L.M. | 2014 | American Journal of Botany, 101 (7), 1167-1175 |
| 22 | *Genetic variation in Aechmea winkleri, a bromeliad from na inland Atlantic rainforest fragment in Southern Brazi* | Goetze, M.; Buttow, M.V.; Zanella, C.M.; Paggi, G.M.; Bruxel, M.; Pinheiro, F.G.; Sampaio, J.A.T.; Palma-Silva, C.; Cidade, F.W. and Bered, F. | 2015 | Biochemical Systematics and Ecology 58, 204-210 |

| Number | Title | Authors | Year | Journal (vol, pages) |
|---|---|---|---|---|
| 23 | *Patterns of Gene Flow in Encholirium horridum L.B.Sm., a Monocarpic Species of Bromeliaceae From Brazil* | Hmeljevski, K.V.; Reis, M.S. and Forzza, R.C. | 2015 | Journal of Heredity 106(1):93–101 |
| 24 | *Clonality strongly affects the spatial genetic structure of the nurse species Aechmea nudicaulis (L.) Griseb. (Bromeliaceae)* | Loh, F.; Scarno, F.R.; Alves-Ferrreira, M. and Salgueiro, F. | 2015 | Botanical Journal of the Linnean Society 178, 329–341. |
| 25 | *Limited pollen flow and high selfing rates toward geographic range limit in an Atlantic forest bromeliad* | Paggi, G.M.; Palma-Silva, C.; Bodanese-Zanetti, M.H.; Lexer, C. and Bered, F. | 2015 | Flora 211, 1–10 |
| 26 | *Mating System variation and assortive mating of sympatric bromeliads (Pitcarinia sp.) endemic to Neotropical inselbergs* | Palma-Silva, C.; Cozzolino, S.; Paggi, G.M.; Lexer, C. and Wendt, T. | 2015 | American Journal of Botany 102 (5), 758-764 |
| 27 | *Development and characterization of microsatellite markers for Vriesea simplex (Bromeliaceae) and cross-amplification in other species of Bromeliaceae* | Neri, J.; Nazareno, A.G.; Wendt, T. and Palma-Silva, C. | 2015 | Biochemical Systematics and Ecology 58, 34e37 |
| 28 | *East-to-west genetic structure in populations of Aechmea calyculata (Bromeliaceae) from the southern Atlantic rainforest of Brazil* | Goetze, M.; Palma-Silva, C.; Zanella, C.M. and Bered, F. | 2016 | Botanical Journal of the Linnean Society 181, 477–490 |
| 29 | *Gene flow and diversification in a species complexo of Alcantarea inserberg bromealids* | Lexer, C.; Marthaler, F.; Humbert, S.; Barbará, T.; Harpe, M.; Bossolini, E.; Paris, M; Martinelli, G. and Versieux, L.M. | 2016 | Botanical Journal of the Linnean Society 181, 505–520. |
| 30 | *Morphological features, nuclear microsatellites and plastid haplotypes reveal hybridisation processes between two sympatric Vriesea species in Brazil (Bromeliaceae)* | Matos, J.Z.; Juan, A.; Agulló, J.C. and Crespo, M.B. | 2016 | Phytotaxa 261,1- 16 |
| 31 | *Microsatellites from Fosterella christophii (Bromeliaceae) by de Novo Transcriptome Sequencing on the Pacific Biosciences RS Platform* | Wohrmann, T.; Huettel, B.; Wagner, N. and Weising, K. | 2016 | Applications in Plant Sciences 4(1) 1500084 |
| 32 | *Hybridization between two sister species of Bromeliaceae: Vriesea carinata and V. incurvata* | Zanella, C.M.; Palma-Silva, C.; Goetze, M. and Bered, F. | 2016 | Botanical Journal of the Linnean Society 181 (3), 491–504 |
| 33 | *Cross-amplification of nuclear microsatellite markers in two species of Cryptanthus Otto & A. Dietr. (Bromeliaceae)* | Ferreira, D.M.C.; Neri, J.; Palma-Silva, C.; Pinangé, D.S.; | 2017 | Braz. J. Bot 40(2), 475–480 |

| Number | Title | Authors | Year | Journal (vol, pages) |
|---|---|---|---|---|
| | | Benko-Iseppon, A.M. and Louzada, R.B. | | |
| 34 | *Population genetic structure of the rock outcrop species Encholirium spectabile (Bromeliaceae): The role of pollination vs. seed dispersal and evolutionary implications* | Gonçalves-Oliveira,R.C.; Wohrmann, T.; Benko-Iseppon, A.M.; Krapp, F.; Alves, M; Wanderley, M.G.L. and Weising, K. | 2017 | American Journal of Botany 104 (6), 868-878 |
| 35 | *Do plant populations on distinct inselbergs talk to each other? A case study of genetic connectivity of a bromeliad species in an Ocbil landscape* | Hmeljevski, K.V.; Nazareno, A.G.; Bueno, M.L.; Reis, M.S. and Forzza, R.C. | 2017 | Ecology and Evolution 7 (13), 4704-4716 |
| 36 | *Natural hybridization and genetic and morphological variation between two epiphytic bromeliads* | Neri, J. Wendt, T. and Palma-Silva, C. | 2017 | AoB PLANTS 10 (1), plx061 |
| 37 | *Cross-amplification and characterization of microsatellite markers in Alcantarea patriae Versieux & Wand.* | Pereira, A.G.; Bernardi, U.C.S; Manhães, V.C.; Ferreira, R.S. and Miranda, F.D. | 2017 | Genetics and Molecular Research 16 (2), gmr16029692 |
| 38 | *Genetic diversity of Bromeliaceae species from the Atlantic Forest* | Sheu, Y.; Cunha-Machado, A.S.; Gontijo, A.B.P.L.; Favoreto, F.C.; Soares, T.C.B. and Miranda, F.D. | 2017 | Genetics and Molecular Research 16 (2), gmr16029636 |
| 39 | *Transferability of nuclear microsatellite markers to the atmospheric bromeliads Tillandsia recurvata and T. aeranthos (Bromeliaceae)* | Chaves, C.J.N.; Aoki-Gonçalves, F.; Leal, B.S.S.; Rossatto, D.R. and Palma-Silva, C. | 2018 | Brazilian Journal of Botany volume 41, 931–935 |
| 40 | *High genetic diversity and moderate genetic structure in the self-incompatible, clonal Bromelia hieronymi* | Godoy, F.M.R.; Lenzi, M.; Ferreira, B.H.S.; Silva, L.V.; Zanella, C.M. and Paggi, G.M. | 2018 | Botanical Journal of the Linnean Society 187 (4), 672–688 |
| 41 | *High genetic diversity and demographic stability in Aechmea kertesziae (Bromeliaceae), a species of sandy coastal plains (restinga habitat) in southern Brazil* | Goetze, M.; Capra, F.; Buttow, M.V.; Zanella, C.M. and Bered, F. | 2018 | Botanical Journal of the Linnean Society 186, 374–388. |
| 42 | *The role of hybridization and introgression in maintaining species integrity and cohesion in naturally isolated inselberg Bromeliads populations* | Mota, M.R.; Pinheiro, F.; Leal, B.S.S.; | 2018 | Plant Biology 21 (1), 122-132 |

| Number | Title | Authors | Year | Journal (vol, pages) |
|---|---|---|---|---|
| | | Wendt, T. and Palma-Silva, C. | | |
| 43 | *Genetic diversity and population structure of Vriesea reitzii (Bromeliaceae), a species from the Southern Brazilian Highlands* | Soares, L.E.; Goetze, M.; Zanella, C.M. and Bered, F. | 2018 | Genetics and Molecular Biology 41 (1)(suppl), 308-317 |
| 44 | *Development, characterization, and transferability of SSR markers for Vriesea carinata (Bromeliaceae) based on RNA sequencing* | Todeschini, C.C.; Parizotto, J.L.; Guzman, F.; Zanella, C.M.; Margis, R.; Goetze, M.; Paggi, G.M.; Costa, L.M., Melo, C.A.; Hirsch, L.D. and Bered, F. | 2018 | Applications in Plant Sciences 6(10), e1184 |
| 45 | *Development of 15 nuclear microsatellite markers in Deuterocohnia (Pitcairnioideae; Bromeliaceae) using 454 pyrosequencing* | Zenk, F.L.; Firmer, C.; Wohrmann, T.; Silva, L.V.; Heising, K.; Huettel, B. and Paggi, G.M. | 2018 | Applications in Plant Sciences 6(4), e1147 |
| 46 | *Ecological niche modeling and a lack of phylogeographic structure in Vriesea incurvata suggest historically stable areas in the southern Atlantic Forest* | Aguiar-Melo, C.; Zanella, C.M.; Goetze, M.; Palma-Silva, C.; Hirsch, L.D.; Neves, B.; Costa, A.F. and Bered, F. | 2019 | American Journal of Botany 106(7), 971–983 |
| 47 | *Contrasting effects of host tree isolation on population connectedness in two tropical epiphytic bromeliads* | Amici, A.A.; Nadkarni, N.M.; DiBlasi, E. and Seger, J. | 2019 | American Journal of Botany 106 (12), 1602-1611 |
| 48 | *Transferability of nuclear microsatellites markers to Vriesea oligantha (Bromeliaceae), an endemic species from Espinhaço Range, Brazil* | Cacossi, T.; Dantas-Queiroz, M.V. and Palma-Silva, C. | 2019 | Brazilian Journal of Botany 42, 727–733 |
| 49 | *Cross-amplifcation of nuclear microsatellite markers in Aechmea distichantha Lem. (Bromeliaceae)* | Godoy, F.M.R.; Paggi, G.M. and Palma-Silva, C. | 2019 | Brazilian Journal of Botany 42, 353–359 |
| 50 | *Dispersal and local persistence shape the genetic structure of a widespread Neotropical plant species with a patchy distribution* | Leal, B.S.S.; Graciano, V.A.; Chaves, C.J.N.; Huacre, L.A.P; | 2019 | Annals of Botany, 124 (3), 499–512 |

| Number | Title | Authors | Year | Journal (vol, pages) |
|---|---|---|---|---|
| | | Heuerts, M. and Palma-Silva, C. | | |
| 51 | *Filogeografia do Complexo Pitcairnia flammea (Bromeliaceae)* | Mota, M.R. | 2019 | Master dissertation - Unesp, Instituto de Biociências, Rio Claro |
| 52 | *Diversidade e Estrutura genética em Aechmea bambusoides (Bromeliaceae), espécie ameaçada de extinção da região Sudeste da Mata Atlântica Brasileira* | Paulo, M.L. (both studies) | 2019 | Master dissertation - Universidade Federal de Viçosa |
| 53 | *Population genetics shed light on species delimitation and life history of the Dyckia pernambucana complex (Bromeliaceae)* | Pinangé, D.S.B.; Louzada, R.B.; Wohrmann, T.; Krapp, F.; Weising, K.; Zizka, G.; Polo, E.M.; Wanderley, M.G. and Benko-Iseppon, A.M. | 2019 | Botanical Journal of the Linnean Society 192 (4) 706–725 |
| 54 | *Microsatellite markers for the endangered Puya raimondii in Peru* | Tumi, L.; Zhang, Y.; Wang, Z.; Suni, M.L.; Burgess, K.S. and Ge, X. | 2019 | Applications in Plant Sciences 7(12), e11308 |
| 55 | *Strong genetic differentiation among populations of Fosterella rusbyi (Bromeliaceae) in Bolivia* | Wohrmann, T.; Michalak, I.; Zizka, G. and Weising, K. | 2019 | Botanical Journal of the Linnean 192(4), 744–759 |
| 56 | *Estudo filogeográfico e hibridação em um grupo de espécies de Vriesea (Bromeliaceae): contribuições para a diversificação do gênero e história evolutiva da Mata Atântica* | Aguiar-Melo, C. | 2020 | PhD thesis – Universidade Federal do Rio Grande do Sul |
| 57 | *High gene flow maintains wide-range species cohesion in Neotropical epiphyte (Tillandsia aeranthos, Bromeliaceae)* | Aoki-Gonçalves, F.; Dantas-Queiroz, M.V.; Guimarães, T.B.; Neffa, V.S. and Palma-Silva, C. | 2020 | Botanical Journal of the Linnean 194 (2), 239–252 |
| 58 | *Population Genetics of Vascular Epiphytes; Optimization of DNA Extraction and Cross Species Transference in Tillandsia usneoides* | Brown, J.I. | 2020 | Master Dissertation - Valdosta State University |
| 59 | *Genetic diversity and reproductive biology of the dioecious and epiphytic bromeliad Aechmea mariae-reginae (Bromeliaceae) in Costa Rica: implications for its conservation* | Cascante-Marín, A.; Trejos, C.; Madrigal, R. and Fuchs, E.J. | 2020 | Botanical Journal of the Linnean Society 192 (4), 773–786 |
| 60 | *Population genetic structure and species delimitation in the Cryptanthus zonatus complex (Bromeliaceae)* | Ferreira, D.M.C.; Palma-Silva, C.; Neri, J.; Medeiros, M.C.M.; Pinangé, D.S.; Benko-Iseppon, A.M. and Louzada, R.B. | 2020 | Master Dissertation - Valdosta State University |

| Number | Title | Authors | Year | Journal (vol, pages) |
|---|---|---|---|---|
| 61 | *Genetic structure and gene fow among populations of Encholirium magalhaesii, a rocky grassland felds bromeliad* | Gonçalves-Oliveira, R.C.; Wohrmann, T.; Weising, K.; Wanderley, M.G. and Benko-Iseppon, A.M. | 2020 | Brazilian Journal of Botany 43, 283–290 |
| 62 | *Interspecific gene flow and an intermediate molecular profile of Dyckia julianae (Bromeliaceae), an endemic species from southern Brazil* | Hirsch, L.D.; Zanella, C.M.; Aguiar-Melo, C.; Costa, L.M.S. and Bered, F. | 2020 | Botanical Journal of the Linnean Society 192, 675–690 |
| 63 | *Transferability of nuclear microsatellite markers to Stigmatodon species: a bromeliad genus endemic to vertical clifs of inselbergs in Brazil* | Manhães, V.C.; Couto, D.R.; Salgueiro, F. and Costa, A.F. | 2020 | Brazilian Journal of Botany 43, 823–830 |
| 64 | *Genetic diversity and karyotype of Pitcairnia azouryi: an endangered species of Bromeliaceae endemic to Atlantic Forest inselbergs* | Manhães, V.C.; Miranda, F.D.; Clarindo, W.R. and Carrijo, T.T. | 2020 | Molecular Biology Reports 47, 179–189 |
| 65 | *From micro- to macroevolution: insights from a Neotropical bromeliad with high population genetic structure adapted to rock outcrops* | Mota, M.R.; Pinheiro, F.; Leal, B.S.S.; Sardelli, C.H.; Wendt, T. and Palma-Silva, C. | 2020 | Heredity 125, 353–370 |
| 66 | *Fire regimes and pollinator behaviour explain the genetic structure of Puya hamata (Bromeliaceae) rosette plants* | Rivadeneira, G.; Ramsay, P.M. and Montúfar, R. | 2020 | Alpine Botany 130, 13–23 |
| 67 | *Strong genetic structure in Dyckia excelsa (Bromeliaceae), an endangered species found on ironstone outcrops in Pantanal, Brazil* | Ruas, R.B.; Paggi, G.M.; Aguiar-Melo, C.; Hirsch, L.D. and Bered, F. | 2020 | Botanical Journal of the Linnean Society 192, 691–705 |
| 68 | *Underlying Microevolutionary processes parallel macroevolutionary patterns in ancient neotropical mountains* | Dantas-Queiroz, M.V.; Cacossi, T.C.; Leal, B.S.S.; Chaves, C.J.N.; Vasconcelos, T.N.C.; Versieux, L.M. and Palma-Silva, C. | 2021 | Journal of Biogeography 48 (9), 2312-2327 |
| 69 | *Comparative phylogeography of bromeliad species: effects of historical processes and mating system on genetic diversity and structure* | Neri, J.; Wendt, T. and Palma-Silva, C. | 2021 | Botanical Journal of the Linnean 197 (2), 263–276 |
| 70 | *Hybridization and species boundaries between three sympatric bromeliads from the Brazilian Atlantic Forest* | Costa, L.M.S., Goetze, M.; Callegari-Jacques, | 2022 | Botanical Journal of the Linnean Society 198 (4), 438–455 |

| Number | Title | Authors | Year | Journal (vol, pages) |
|---|---|---|---|---|
| | | S.M.; Hirsch, L.D. and Bered, F. | | |
| 71 | *Spatiotemporal Variation on Fertility, Mating System, and Gene Flow in Vriesea gigantea (Bromeliaceae), an Atlantic Forest Species* | Paggi, G.M.; Palma-Silva, C.; Zanella, C.M.; Goetze, M.; Buttow, M.V.; Lexer, C. and Bered, F. | 2022 | Frontiers For. Glob. Change 5, 893548 |
| 72 | *Genetic diversity and genetic structure of Puya raimondii (Bromeliaceae) for its conservation in the Peruvian Andes* | Tumi, L.; Ge, X.; Prado, G.E.; Cosacov, A.; Garcia, V.H.; Arakaki, M. and Suni, M.L. | 2022 | Revista peruana de biología 29(2), e22557 |
| 73 | *Patchily distributed but not necessarily isolated populations of Deuterocohnia meziana: a threatened bromeliad from rock outcrops* | Vicente-Silva, L.; Godoy, F.M.R.; Faggioni, G.P.; Lorenz, A.P. and Paggi, G.M. | 2022 | Botanical Journal of the Linnean Society 199 (1), 312–330 |
| 74 | *Genetic diversity and population structure of Aechmea distichantha (Bromeliaceae), a widely geographically distributed species in South America* | Godoy, F.M.R.; Vicente-Silva, L.; Dantas-Queiroz, M.V.; Palma-Silva, C. and Paggi, G.M. | 2023 | Plant Syst Evol 309, 5 |
| 75 | *Surrounded by concrete: genetic isolation of Tillandsia recurvata L. in an urban landscape in southeastern Brazil* | Quail, M.R.; Ramos, F.N.; Dallimore, T.; Ashton, P.; Clayton-Brown, J.; Provan, J. and Batke, S.P. | 2023 | Botanical Journal of the Linnean Society, 2023; boad 31 https://doi.org/10.1093/botlinnean/boad031 |
