# Supplementary material for: Genetic diversity and conservation in Bromeliaceae based on SSR markers
Source: Genet Mol Biol. 2024 Apr 26;46(3 Suppl 1):e20230135. doi: 10.1590/1678-4685-GMB-2023-0135 (PMC11113272; doi:10.1590/1678-4685-GMB-2023-0135)
Supplement: Table S2 - [file 1415-4757-GMB-46-03-s1-e20230135-s2.pdf]

Genetics and Molecular Biology

https://doi.org/10.1590/1678-4685-GMB-2023-0135

Supplementary Material to "Genetic diversity and conservation in Bromeliaceae based on SSR markers"

Table S2 - Total of sampled taxa or each subfamily in the 75 studies with microsatellites analyzed in this article.

Bromelioideae Subfamily	Pitcairnioideae Subfamily	Tillandsioideae Subfamily	Puyoideae Subfamily
Aechmea bambusoides L.B.Sm. & Reitz	*Deuterocohnia brevispicata* Rauh & L.Hrom.	*Alcantarea brasiliana* (L.B.Sm.) J.R.Grant	*Puya hamata* L.B. Sm.
Aechmea calyculata (É.Morren) Baker	*Deuterocohnia brevifolia* (Griseb.) M.A.Spencer & L.B.Sm.	*Alcantarea geniculata* (Wawra) J.R.Grant	*Puya hutchisonii* L.B.Sm.
Aechmea caudata Lindm.	*Deuterocohnia longipetala* (Baker) Mez	*Alcantarea glaziouana* (Lem.) Leme	*Puya macrura* Mez
Aechmea comata (Gaudich.) Baker	*Deuterocohnia meziana* Kuntze ex Mez	*Alcantarea imperialis* (Carrière) Harms	*Puya macropoda* L.B.Sm.
Aechmea distichantha Lem.	*Deuterocohnia meziana* subsp. *Carmineoviridiflora* Rauh.	*Alcantarea martinellii* Versieux & Wand.	*Puya raimondii* Harms
Aechmea kertesziae Reitz	*Deuterocohnia meziana* subsp. m*eziana* Kuntze ex Mez.	*Alcantarea nevaresii* Leme	
Aechmea mariae-reginae H. Wendl	*Deuterocohnia seramisiana* R.Vásquez, Ibisch & E.Gross	*Alcantarea patriae* Versieux & Wand.	
Aechmea nudicaulis (L.) Griseb.	*Dyckia choristaminea* Mez	*Alcantarea regina* (Vell.) Harms	
Aechmea ramosa Mart. ex Schult. & Schult.f.	*Dyckia dissitiflora* Schult. & Schult.f.	*Catopsis nitida* (Hook.) Griseb	
Aechmea winkleri Reitz	*Dyckia distachya* Hassler	*Guzmania monostachia* (L.) Rusby ex Mez	
Ananas comosus (L.) Merr.	*Dyckia excelsa* Leme	*Stigmatodon brassicoides* (Baker) Leme, G. K. Br. & Barfuss	
Billbergia euphemiae E.Morren	*Dyckia hebdingii* L.B.Sm.	*Stigmatodon costae* (B. R. Silva & Leme) Leme, G. K. Br. & Barfuss	
Billbergia horrida Regel	*Dyckia julianae* Strehl	*Stigmatodon goniorachis* (Baker) Leme, G. K. Br. & Barfuss	
Bromelia antiacantha Bertol.	*Dyckia limae* L.B. Sm.	*Stigmatodon sp.*	
Bromelia hieronymi Mez	*Dyckia pernambucana* L.B. Sm.	*Tillandsia aeranthos* (Loisel.) Desf.	
Cryptanthus burle-marxii Leme	*Encholirium horridum* S.B.Sm.	*Tillandsia recurvata* (L.) L.	
Cryptanthus zonatus (Vis.) Beer	*Encholirium magalhaesii*	*Tillandsia usneoides* (L.) L.	

Bromelioideae Subfamily	Pitcairnioideae Subfamily	Tillandsioideae Subfamily	Puyoideae Subfamily
	L.B.Sm.		
Orthophytum ophiuroides Louzada & Wand.	*Encholirium spectabile* Mart. ex Schult. & Schult.f.	*Vriesea carinata* Wawra	
	Fosterella christophii Ibisch, R. Vásquez & J. Peters	*Vriesea gigantea* Gaudich.	
	Fosterella rusbyi (Mez) L.B.Sm.	*Vriesea incurvata* Gaudich.	
	Pitcairnia albiflos Herb.	*Vriesea inflata* Wawra (Wawra)	
	Pitcairnia azouryi Martinelli & Forzza	*Vriesea minarum* L.B.Sm.	
	Pitcairnia carinata Mez	*Vriesea oligantha* (Baker) Mez	
	Pitcairnia corcovadensis Wawra	*Vriesea reitzii* Leme & A.F.Costa	
	Pitcairnia curvidens L. B. Sm. & Read	*Vriesea scalaris* É.Morren	
	Pitcairnia flammea Lindl.	*Vriesea simplex* (Vell.) Beer	
	Pitcairnia flammea Lindl complex	*Vriesea taritubensis* var. *patens* B. Neves & A.F. Costa	
	Pitcairnia flammea var. *flammea* Lindl.	*Werauhia tonduziana* (L.B.Sm.) J.R.Grant	
	Pitcairnia flammea var. *floccosa* L.B. Sm.		
	Pitcairnia flammea var. *macropoda* L.B.Sm. & Reitz		
	Pitcairnia flammea var. *pallida* L.B.Sm.		
	Pitcairnia flammea var. *roeltzii* (E.Morren) L.B.Sm.		
	Pitcairnia geyskesii L.B.Sm.		
	Pitcairnia lanuginosa Ruiz & Pav.		
	Pitcairnia spp.		
	Pitcairnia staminea G.Lodd.		
**Total Bromelioideae 18**	**Total Pitcairnioideae: 36**	**Total Tillandsioideae: 28**	**Total Puyoideae: 5**
	**Total: 87**		
