# Supplementary material for: Genetic diversity and conservation in Bromeliaceae based on SSR markers
Source: Genet Mol Biol. 2024 Apr 26;46(3 Suppl 1):e20230135. doi: 10.1590/1678-4685-GMB-2023-0135 (PMC11113272; doi:10.1590/1678-4685-GMB-2023-0135)
Supplement: Table S3 - [file 1415-4757-GMB-46-03-s1-e20230135-s3.pdf]

Genetics and Molecular Biology

https://doi.org/10.1590/1678-4685-GMB-2023-0135

Supplementary Material to "Genetic diversity and conservation in Bromeliaceae based on SSR markers"

Table S3 - Subfamilies, taxa, number of populations (N pops), sampled individuals (N ind), nuclear microsatellite markers used (N SSRs), average of genetic diversity parameters (Allelic Richness - AR, *Ho*, H_E, F_{IS}, F_{ST}), Mating System, Seed Dispersal Mechanism, Pollination Syndrome, Biome, National Conservation (NC) and Local Conservation (LC) Status for each taxa analyzed in the 75 studies considered in the present work. Acronyms: BA (Bahia); ES (Espírito Santo); MG (Minas Gerais); MS (Mato Grosso do Sul); PB (Paraíba); PE (Pernambuco); PR (Paraná); RJ (Rio de Janeiro); RN (Rio Grande do Norte); RS (Rio Grande do Sul); SC (Santa Catarina); SE (Sergipe); SP (São Paulo); AR (Argentina); BO (Bolivia); CO (Colombia); CR (Costa Rica); EC (Ecuator); GF (French Guiana); MX (Mexico); PE (Peru); US (United States). NA: Not Threatened; DD: Data Deficient; LC: Least Concern; NT: Near Threatened; VU: Vulnerable; EN: Endangered; CR: Critically Endangered. – no data available. The last column corresponds to the reference number indicated in Table S3.

Subfamily/ Taxa	N pops	N ind	N SSRs	RA	*Ho*	*He*	F_{IS}	F_{ST}	Mating System	Seed Dispersal	Pollination	Biome	NC	LC	Reference	Number
**Bromelioideae**																
Aechmea bambusoides L.B.Sm. & Reitz	7	92	8	1.253	0.061	0.422	0.855	0.269	Mixed	Anemochory	Ornitophily	Atlantic Forest (RJ, MG)	VU	EN	Paulo *et al.*, 2019	52
Aechmea bambusoides L.B.Sm. & Reitz	1	17	6	-	0.035	0.299	0.770	-	Mixed	Anemochory	Ornitophily	Atlantic Forest (RJ, MG)	VU	EN	Paulo *et al.*, 2019	52
Aechmea calyculata (E.Morren) Baker	5	14	12	3.78	0.505	0.576	0.104	0.218	Mixed or Alogamous	Zoochory	Ornitophily	Atlantic Forest (RS, SC)	VU	CR	Goetze *et al.*, 2016	28
Aechmea caudata Lindm.	3	54	10	1.772	0.319	0.389	0.159	-	Alogamous	Zoochory	Entomophily	Atlantic Forest (SC)	LC	EN	Costa *et al.*, 2022	70
Aechmea caudata Lindm.	2	37	10	3.122	0.482	0.657	0.262	-	Alogamous	Zoochory	Entomophily	Atlantic Forest (SC)	LC	EN	Goetze *et al.*, 2013	15
Aechmea comata (Gaudich.) Baker	6	119	10	2.601	0.678	0.686	-0.0025		Mixed	Zoochory	Ornitophily, Entomophily	Atlantic Forest (SC)	-	-	Costa *et al.*, 2022	70

Subfamily/ Taxa	N pops	N ind	N SSRs	RA	*Ho*	*He*	F_{IS}	F_{ST}	Mating System	Seed Dispersal	Pollination	Biome	NC	LC	Reference	Number
Aechmea distichantha Lem.	3	47	10	3.005	0.529	0.624	0.153	0.210	Mixed with Clonal Reproduction	Zoochory	Ornitophily, Entomophily	Cerrado (SP, MS)	LC	VU	Godoy *et al.*, 2019	49
Aechmea distichantha Lem.	7	137	10	2.692	0.366	0.544	0.252	0.240	Mixed with Clonal Reproduction	Zoochory	Ornitophily, Entomophily	Cerrado/ Atlantic Forest (RJ, MG, SP, MS, PR, SC, RS)	LC	VU	Godoy *et al.*, 2023	74
Aechmea kertesziae Reitz	3	51	10	2.272	0.583	0.564	-0.013	-	Alogamous with Clonal Reproduction	Zoochory	Entomophily	Atlantic Forest (SC)	EN	EN	Costa *et al.*, 2022	70
Aechmea kertesziae Reitz	4	103	9	6.125	0.625	0.715	0.146	0.093	Alogamous with Clonal Reproduction	Zoochory	Entomophily	Atlantic Forest (SC)	EN	EN	Goetze *et al.*, 2018	41
Aechmea mariae-reginae H.Wendl.	6	106	8	5.238	0.540	0.637	0.123	0.180	Alogamous	Zoochory	Ornitophily	American Continent (CR)	-	-	Cascante-Marín *et al.*, 2020	59
Aechmea nudicaulis (L.) Griseb.	-	249	6	-	0.690	0.710	0.040		Alogamous with Clonal Reproduction	Zoochory	Ornitophily	Atlantic Forest (RJ)	LC	VU	Loh *et al.*, 2015	24
Aechmea nudicaulis (L.) Griseb.	-	8	10	-	0.48	0.50	-	-	Alogamous with Clonal Reproduction	Zoochory	Ornitophily	Atlantic Forest (ES)	LC	VU	Miranda *et al.*, 2012	12
Aechmea nudicaulis (L.) Griseb.	5	35	5	3.16	0.576	0.519	-0.110		Alogamous with Clonal Reproduction	Zoochory	Ornitophily	Atlantic Forest (ES)	LC	VU	Sheu *et al.*, 2017	38
Aechmea ramosa Mart. ex Schult. & Schult.f.	-	12	10	-	0.470	0.480	-	-	-	Zoochory	Ornitophily	Atlantic Forest (ES)	-	NA	Miranda *et al.*, 2012	12
Aechmea ramosa Mart. ex Schult. & Schult.f.	5	32	8	3.12	0.407	0.481	0.143	-	-	Zoochory	Ornitophily	Atlantic Forest (ES)	-	NA	Sheu *et al.*, 2017	38
Aechmea winkleri Reitz	4	162	7	3.31	0.563	0.582	0.043	0.05	Alogamous with Clonal Reproduction	Zoochory	Entomophily	Atlantic Forest (RS)	VU	EN	Goetze *et al.*, 2015	22
Billbergia euphemiae E.Morren	-	4	20	-	0.67	0.47	-	-	Alogamous	Zoochory	Ornitophily	Atlantic Forest (ES)	-	NA	Miranda *et al.*, 2012	12
Billbergia euphemiae E.Morren	5	26	9	3.00	0.390	0.483	0.177	-	Alogamous	Zoochory	Ornitophily	Atlantic Forest (ES)	-	NA	Sheu *et al.*, 2017	38
Billbergia horrida Regel	-	10	10	-	0.55	0.46	-	-	Alogamous	Zoochory	Ornitophily	Atlantic Forest (ES)	-	NA	Miranda *et al.*, 2012	12
Billbergia horrida Regel	5	31	7	3.00	0.430	0.435	-0.041		Alogamous	Zoochory	Ornitophily	Atlantic Forest (ES)	-	NA	Sheu *et al.*, 2017	38

Subfamily/ Taxa	N pops	N ind	N SSRs	RA	*Ho*	*He*	F_{IS}	F_{ST}	Mating System	Seed Dispersal	Pollination	Biome	NC	LC	Reference	Number
Bromelia antiacantha Bertol.	7	165	5	3.95	0.328	0.550	0.400	0.127	Mixed with Clonal Reproduction	Zoochory	Ornitophily, Chiropterophily	Atlantic Forest (RS, SC)	DD	LC	Zanella *et al*., 2011	11
Bromelia hieronymi Mez.	3	154	5	3.27	0.624	0.558	-0.094	0.138	Alogamous with Clonal Reproduction	Zoochory	Ornitophily, Entomophily	Pantanal (MS)	-	-	Godoy *et al*., 2018	40
Cryptanthus burle-marxii Leme	5	24	10	3.287	0.440	0.438	-0.031	-	Alogamous	Zoochory	Vertebrates	Atlantic Forest (RN, PE)	VU	-	Ferreira *et al*., 2017	33
Cryptanthus zonatus (Vis.) Beer	7	14	-	2.8	0.458	0.509	0.127	-	Alogamous	Zoochory	Vertebrates	Atlantic Forest (PE)	VU	-	Ferreira *et al*., 2017	33
Cryptanthus zonatus (Vis.) Beer Complex	8	106	10	1.822	0.497	0.463	-0.010	0.121	Alogamous	Zoochory	Vertebrates	Atlantic Forest (RN, PE)	VU	-	Ferreira *et al*., 2020	60
Orthophytum ophiuroides Louzada & Wand.	2	44	11	-	0.712	0.623	0.223	-	-	Anemochory	Entomophily	Caatinga (BA)	-	-	Aoki-Gonçalvez *et al*., 2014	19
**Pitcairnioideae**																
Deuterocohnia brevispicata Rauh & L.Hrom.	1	13	15	-	0.49	0.62	0.27	-	Alogamous	Anemochory, Hydrochory or Accidental Zoochory	Ornitophily, Entomophily, Chiropterophily	American Continent (BO)	-	-	Zenk *et al.*, 2018	45
Deuterocohnia longipetala (Baker) Mez	1	23	15	-	0.44	0.66	0.32	-	Alogamous	Anemochory, Hydrochory or Accidental Zoochory	Ornitophily, Entomophily	American Continent (AR, BO)	-	-	Zenk *et al.*, 2018	45
Deuterocohnia meziana Kuntze ex Mez	6	114	8	3.631	0.566	0.526	-0.069	0. 165	Alogamous	Anemochory	Ornitophily, Entomophily	Pantanal (MS)	VU	-	Vicente-Silva *et al.*, 2022	73
Deuterocohnia meziana subsp. *carmineoviridiflora* Rauh.	2	28	15	-	0.47	0.52	0.096	-	Alogamous	Anemochory	Ornitophily, Entomophily, Chiropterophily	American Continent (BO)	EN	-	Zenk *et al.*, 2018	45
Deuterocohnia meziana subsp. *Meziana* Kuntze ex Mez.	5	48	15	-	0.22	0.35	0.24	-	Alogamous with Clonal Reproduction	Anemochory	Ornitophily, Entomophily, Chiropterophily	Pantanal (MS)	EN	-	Zenk *et al.*, 2018	45
Deuterocohnia seramisiana R.Vásquez, Ibisch & E.Gross	1	17	15	-	0.54	0.58	0.08	-	Alogamous	Anemochory, Hydrochory or Accidental Zoochory	Entomophily, Chiropterophily	American Continent (BO)	-	-	Zenk *et al.*, 2018	45
Dyckia choristaminea Mez	3	78	7	2.765	0.480	0.564	0.153	-	-	Anemochory	-	Pampa (RS)	DD	EN	Hirsch *et al.*, 2020	62

Subfamily/ Taxa	N pops	N ind	N SSRs	RA	*Ho*	*He*	F_{IS}	F_{ST}	Mating System	Seed Dispersal	Pollination	Biome	NC	LC	Reference	Number
Dyckia dissitiflora Schult. & Schult.f	3	37	15	4.529	0.452	0.553	0.161	-	-	Anemochory	Ornitophily	-	LC	-	Pinangé *et al.*, 2019	53
Dyckia dissitiflora Schult. & Schult.f	2	30	15	-	0.428	0.506	-	-	-	Anemochory	Ornitophily	-	LC		Wohrmann *et al.*, 2013	18
Dyckia distachya Hassler	1	21	9	-	0.241	0.419	-	-	Alogamous with Clonal Reproduction	Anemochory	Ornitophily, Entomophily	Atlantic Forest (RS)	CR	EN	Zanella *et al.*, 2012	14
Dyckia excelsa Leme	7	101	7	1.764	0.251	0.396	0.395	0.437	Mixed with Clonal Reproduction	Anemochory	Ornitophily, Entomophily	Pantanal (MS)			Ruas *et al.*, 2020	67
Dyckia hebdingii L.B.Sm.	4	117	7	1.9	0.326	0.382	0.138		Autogamous	Anemochory	-	Pampa (RS)	DD	VU	Hirsch *et al.*, 2020	62
Dyckia julianae Strehl	2	49	7	2.9	0.515	0.637	1.68	-	-	Anemochory	-	Pampa (RS)	DD	VU	Hirsch *et al.*, 2020	62
Dyckia limae L.B.Smith	1	10	15	3.350	0.380	0.391	0.010	-	-	Anemochory	Ornitophily	-	-	-	Pinangé *et al.*, 2019	53
Dyckia limae L.B.Smith	1	10	15	-	0.38	0.39	-	-	-	Zoochory	Vertebrates	-	-	-	Wohrmann *et al.*, 2013	18
Dyckia pernambucana L.B.Smith	4	40	15	2.556	0.358	0.356	0.276	-	Autogamous	Anemochory	Ornitophily	-			Pinangé *et al.*, 2019	53
Dyckia pernambucana L.B.Smith	2	19	15	-	0.222	0.244	-	-	Autogamous	Zoochory	Vertebrates	-	-	-	Wohrmann *et al.*, 2013	18
Encholirium horridum S.B.Sm.	11	89	10	-	0.336	0.429	-	-	Alogamous with Clonal Reproduction	Anemochory	Ornitophily, Entomophily, Chiropterophily	Atlantic Forest (BA, MG, ES, RJ)	CR	VU	Hmeljevski *et al.*, 2013	16
Encholirium horridum S.B.Sm.	1	304	7	-	0.809	0.849	0.046	-	Alogamous with Clonal Reproduction	Anemochory	Ornitophily, Entomophily, Chiropterophily	Atlantic Forest (ES)	CR	VU	Hmeljevski *et al.*, 2015	24
Encholirium horridum S.B.Sm.	11	526	8	5.26	-	0.464	0.205	-	Alogamous with Clonal Reproduction	Anemochory	Ornitophily, Entomophily, Chiropterophily	Atlantic Forest (BA, RJ, MG, ES)	CR	VU	Hmeljevski *et al.*, 2017 I	35
Encholirium magalhaesii L.B.Sm.	4	39	4	6.494	0.424	0.533	0.194	0.197	-	Anemochory	Chiropterophily	Atlantic Forest (MG)	-	-	Gonçalves-Oliveira *et al.*, 2020	61
Encholirium spectabile Mart. ex Schult. & Schult.f.	20	196	8	3.159	0.497	0.656	0.256	0.219	-	Anemochory	Ornitophily, Chiropterophily	Caatinga (BA, PB, PE, SE)	LC	-	Gonçalves-Oliveira *et al.*, 2017	34

Subfamily/ Taxa	N pops	N ind	N SSRs	RA	*Ho*	*He*	F_{IS}	F_{ST}	Mating System	Seed Dispersal	Pollination	Biome	NC	LC	Reference	Number
Fosterella christophii Ibisch, R. Vásquez & J. Peters	3	29	13	-	0.415	0.568	-	-	Autogamous	Anemochory	Entomophily	American Continent (BO)	-	-	Wohrmann *et al.*, 2016	31
Fosterella rusbyi (Mez) L.B.S	7	30	15	-	0.135	0.467	0.746	-	Autogamous	Anemochory	Entomophily	American Continent (BO)	-	-	Wohrmann *et al.*, 2012	13
Fosterella rusbyi (Mez) L.B.S	30	253	15	1.145	0.089	0.143	0.326	-	Mixed with Clonal Reproduction	Anemochory	Entomophily	American Continent (BO)	-	-	Wohrmann *et al.*, 2019	55
Pitcairnia albiflos Herb.	1	22	8	-	0.408	0.663	-	-	Alogamous	Anemochory	Entomophily, Chiropterophily	Atlantic Forest (RJ)	CR	VU	Paggi *et al.*, 2008	5
Pitcairnia albiflos Herb.	3	-	6	-	-	-	0.063	-	Alogamous	Anemochory	Entomophily, Chiropterophily	Atlantic Forest (RJ)	CR	VU	Palma-Silva *et al.*, 2015	25
Pitcairnia albiflos Herb.	5	134	15	3.20	0.383	0.428	0.109	0.336	Alogamous	Anemochory	Entomophily, Chiropterophily	Atlantic Forest (RJ)	CR	VU	Palma-Silva *et al.*, 2011	10
Pitcairnia azouryi Martinelli and Forzza	5	66	9	3.221	0.470	0.519	0.130	0.188	Alogamous with Clonal Reproduction	Anemochory	-	Atlantic Forest (RJ, ES)	-	EN	Manhães *et al.*, 2020	64
Pitcairnia carinata Mez.	1	20	10	4.208	0.528	0.685	0.289	-	-	-	-	Atlantic Forest (RJ)	-	DD	Mota *et al.*, 2019	51
Pitcairnia corcovadensis Wawra	2	32	8	1.959	0.381	0.455	0.150	-	Autogamous	Anemochory	Ornitophily	Atlantic Forest (RJ)	VU	VU	Mota *et al.*, 2018	42
Pitcairnia corcovadensis Wawra	1	20	10	5.610	0.524	0.789	0.352	-	Autogamous	Anemochory	Ornitophily	Atlantic Forest (RJ)	VU	VU	Mota *et al.*, 2019	51
Pitcairnia curvidens L. B. Sm. & ReaD	1	20	10	5.268	0.709	0.687	-0.059	-	-	-	-	Atlantic Forest (MG)	-	NT	Mota *et al.*, 2019	51
Pitcairnia flammea Lindl.	-	12	10	-	0.31	0.42	-	-	Mixed	Zoochory	Ornitophily	Atlantic Forest (ES)	LC	-	Miranda *et al.*, 2012	12
Pitcairnia flammea Lindl complex	25	501	10	3.658	0.464	0.550	0.154	0.374	Mixed	Zoochory	Ornitophily	Atlantic Forest (RJ, SP, MG, ES, PR)	LC	-	Mota, 2020	65
Pitcairnia flammea var. *flammea* Lindl.	12	254	10	3.314	0.399	0.457	0.156	-	Mixed	Zoochory	Ornitophily	Atlantic Forest (RJ, SP, MG, ES)	LC	-	Mota *et al.*, 2019	51
Pitcairnia flammea var. *floccosa* L.B. Sm.	5	114	10	3.947	0.495	0.613	0.194	-	Mixed	Zoochory	Ornitophily	Atlantic Forest (SP, MG, PR)	LC	-	Mota *et al*, 2019	51

Subfamily/ Taxa	N pops	N ind	N SSRs	RA	*Ho*	*He*	F_{IS}	F_{ST}	Mating System	Seed Dispersal	Pollination	Biome	NC	LC	Reference	Number
Pitcairnia flammea var. *macropoda* L.B.Sm. & Reitz	2	41	10	3.799	0.545	0.608	0.08	-	Mixed	Zoochory	Ornitophily	Atlantic Forest (MG, RJ)	LC	-	Mota *et al.*, 2019	51
Pitcairnia flammea var. *pallida* L.B.Sm.	1	12	10	4.144	0.673	0.644	-0.060	-	Mixed	Zoochory	Ornitophily	Atlantic Forest (RJ)	LC	-	Mota *et al.*, 2019	51
Pitcairnia flammea var. *roeltzii* (E.Morren) L.B.Sm.	1	20	10	2.555	0.313	0.358	0.111	-	Mixed	Zoochory	Ornitophily	Atlantic Forest (RJ)	LC	-	Mota *et al.*, 2019	51
Pitcairnia geyskesii L.B.Sm.	14	413	7	2.65	0.293	0.325	0.092	0.533	-	Anemochory	Ornitophily	American Continent (FG)	EN	-	Boisselier-Dubayle *et al.*, 2010	8
Pitcairnia geyskesii L.B.Sm.	5	40	7	-	0.288	0.301	-	-	-	Anemochory	Ornitophily	American Continent (FG)	EN	-	Sarthou *et al.*, 2003	1
Pitcairnia lanuginosa Ruiz & Pav.	22	318	8	-	0.094	0.204	0.502	0.73	Autogamous	-	-	Cerrado (RJ, MG, ES, MT, MS, GO, TO, PA); American Continent (PE)	-	-	Leal *et al.*, 2019	50
Pitcairnia sp.	1	7	10	2.599	0.509	0.479	-0.090	-	-	-	-	Atlantic Forest (SP)	-	-	Mota *et al.*, 2019	51
Pitcairnia staminea G.Lodd.	4	119	15	3.71	0.347	0.452	0.239	0.282	Autogamous	Anemochory	Entomophily	Atlantic Forest (RJ)	-	EN	Palma-Silva *et al.*, 2011	10
Pitcairnia staminea G.Lodd.	3	-	2	-	-	-	0.193	-	Autogamous	Anemochory	Entomophily	Atlantic Forest (RJ)	-	EN	Palma-Silva *et al.*, 2015	25
**Tillandsioideae**																
Alcantarea brasiliana (L.B.Sm.) J.R.Grant	7	134	9	2.467	0.240	0.34	-	-	-	Anemochory	Entomophily	Atlantic Forest (RJ)	-	-	Lexer *et al.*, 2016	30
Alcantarea geniculata (Wawra) J.R.Grant	4	168	7	2.31	0.347	0.368	0.075	-	Alogamous	Anemochory	Entomophily	Atlantic Forest (RJ)	LC	EN	Barbará *et al.*, 2007	2
Alcantarea geniculata (Wawra) J.R.Grant	1	32	9	-	-	0.357	0.192	-	Alogamous	Anemochory	Entomophily	Atlantic Forest (RJ)	LC	EN	Barbará *et al.*, 2008	4
Alcantarea geniculata (Wawra) J.R.Grant	4	84	8	5.250	0.357	0.429	-	-	Alogamous	Anemochory	Entomophily	Atlantic Forest (RJ)	LC	EN	Barbará *et al.*, 2009	6
Alcantarea geniculata (Wawra) J.R.Grant	7	64	9	3.007	0.348	0.43	-	-	Alogamous	Anemochory	Entomophily	Atlantic Forest (RJ)	LC	EN	Lexer *et al.*, 2016	30

Subfamily/ Taxa	N pops	N ind	N SSRs	RA	*Ho*	*He*	F_{IS}	F_{ST}	Mating System	Seed Dispersal	Pollination	Biome	NC	LC	Reference	Number
Alcantarea glaziouana (Lem.) Leme	4	99	9	-	-	0.415	0.247	-	Clonal Reproduction	Anemochory	Chiropterophily	Atlantic Forest (RJ)	LC	EN	Barbará *et al.*, 2008	4
Alcantarea glaziouana (Lem.) Leme	5	170	8	1.95	0.259	0.333	0.156	0.246	Clonal Reproduction	Anemochory	Chiropterophily	Atlantic Forest (RJ)	LC	EN	Barbará *et al.*, 2009	6
Alcantarea imperialis (Carrière) Harms	4	248	8	2.48	0.357	0.398	-0.0006		Mixed	Anemochory	Ornitophily, Entomophily, Chiropterophily	Atlantic Forest (RJ)	LC	VU	Barbará *et al.*, 2007	2
Alcantarea imperialis (Carrière) Harms	2	135	9	-	-	0.393	0.061	-	Mixed		Ornitophily, Entomophily, Chiropterophily	Atlantic Forest (RJ, MG)	LC	VU	Barbará *et al.*, 2008	4
Alcantarea imperialis (Carrière) Harms	4	124	8	6.250	0.362	0.615	-	-	Mixed	Anemochory	Ornitophily, Entomophily, Chiropterophily	Atlantic Forest (RJ)	LC	VU	Barbará *et al.*, 2009	6
Alcantarea imperialis (Carrière) Harms	7	54	9	3.476	0.402	0.48	-	-	Mixed	Anemochory	Ornitophily, Entomophily, Chiropterophily	Atlantic Forest (RJ)	LC	VU	Lexer *et al.*, 2016	30
Alcantarea imperialis (Carrière) Harms	4	30	4	-	0.359	0.371	-	-	Mixed	Anemochory	Ornitophily, Entomophily, Chiropterophily	Atlantic Forest (RJ)	LC	VU	Palma-Silva *et al.*, 2007	3
Alcantarea martinellii Versieux & Wand.	7	20	9	1.774	0.356	0.271	-	-	-	Anemochory	Vertebrates	Atlantic Forest (RJ)	CR	VU	Lexer *et al.*, 2016	30
Alcantarea nevaresii Leme	7	46	9	1.848	0.206	0.217	-	-	-	Anemochory	Vertebrates	Atlantic Forest (RJ)	CR	VU	Lexer *et al.*, 2016	30
Alcantarea patriae Versieux & Wand.	1	20	12	4.586	0.461	0.659	0.344	-	-	Anemochory	Entomophily	Atlantic Forest (ES)	NT	-	Pereira *et al.*, 2017	37
Alcantarea regina (Vell.) Harms	2	44	9	-	-	0.464	-0.044	-	-	Anemochory	Ornitophily, Entomophily, Chiropterophily	Atlantic Forest (RJ)	-	-	Barbará *et al.*, 2008	4
Alcantarea regina (Vell.) Harms	2	55	8	3.496	0.479	0.458	-0.051	-	-	Anemochory	Ornitophily, Entomophily, Chiropterophily	Atlantic Forest (RJ)	-	-	Barbará *et al.*, 2009	6
Catopsis nitida (Hook.) Griseb	17	265	8	-	0.104	0.201	0.296	0.26	Autogamous	Anemochory	Entomophily	American Continent (CR)	-	-	Amici *et al.*, 2019	47
Guzmania monostachia (L.) Rusby ex Mez	18	364	6	-	0.028	0.359	0.917	-	Mixed	Anemochory	Ornitophily	American Continent (CR)	DD	-	Cascante-Marín *et al.*, 2014	20
Stigmatodon brassicoides (Baker)	3	-	10	-	0.297	0.342	-	-	-	-	-	Atlantic Forest (RJ)	EN	-	Manhães *et al.*, 2020	63

**Subfamily/ Taxa**	**N pops**	**N ind**	**N SSRs**	**RA**	***Ho***	***He***	F_{IS}	F_{ST}	**Mating System**	**Seed Dispersal**	**Pollination**	**Biome**	**NC**	**LC**	**Reference**	**Number**
Leme, G. K. Br. & Barfuss																
Stigmatodon costae (B. R. Silva & Leme) Leme, G. K. Br. & Barfuss	7	-	10	-	0.405	0.445	-	-	-	-	-	Atlantic Forest (RJ)	CR	-	Manhães *et al.*, 2020	63
Stigmatodon goniorachis (Baker) Leme, G. K. Br. & Barfuss	7	-	12	-	0.433	0.488	-	-	-	-	-	Atlantic Forest (RJ)	EN	-	Manhães *et al.*, 2020	63
Stigmatodon sp.	7	-	12	-	0.312	0.354	-	-	-	-	-	Atlantic Forest (RJ)	-	-	Manhães *et al.*, 2020	63
Tillandsia aeranthos (Loisel.) Desf.	2	37	7	7.736	0.756	0.857	0.115	0.0128	Alogamous	Anemochory	Ornitophily	Pampa (RS)	-	VU	Chaves *et al.*, 2018	39
Tillandsia aeranthos (Loisel.) Desf.	13	203	7	5.678	0.745	0.751	0.163	0.031	Alogamous	Anemochory	Ornitophily	Pampa (RS); American Continent (AR)	-	VU	Aoki-Gonçalves *et al.*, 2020	57
Tillandsia recurvata (L.) L.	-	90	5	9.51	0.279	0.605	0.44	-	Autogamous	Anemochory	Entomophily	American Continent (MX)	-	LC	Solorzano *et al.*, 2010	9
Tillandsia recurvata (L.) L.	2	41	7	5.894	0.068	0.629	0.803	0.839	Autogamous	Anemochory	Entomophily	Cerrado (SP)	-	LC	Chaves *et al.*, 2018	39
Tillandsia recurvata (L.) L.	65	288	13	3.913	0.346	0.673	0.482	-	Autogamous	Anemochory	Entomophily	(MG)	-	LC	Quail *et al.*, 2023	75
Tillandsia usneoides (L.) L.	-	35	7	-	0.394	0.435	-0.028	-	Clonal Reproduction	-	-	American Continent (US)	LC	VU	Brown *et al.*, 2020	58
Vriesea carinata Wawra	-	20	3	-	0.727	0.812	0.212		Autogamous	Anemochory	Ornitophily	Atlantic Forest (SC)	LC	VU	Matos *et al.*, 2016	30
Vriesea carinata Wawra	6	38	14	4.95	0.518	0.675	0.237	-	Autogamous	Anemochory	Ornitophily	Atlantic Forest (SP, RS, SC, PR)	LC	VU	Zanella *et al.*, 2016	32
Vriesea carinata Wawra	4	69	16	-	0.346	0.591	0.383	-	Autogamous	Anemochory	Ornitophily	Atlantic Forest (PR, SP)	LC	VU	Todeschini *et al.*, 2018	44
Vriesea carinata Wawra	2	59	12	3.544	0.412	0.559	0.258	-	Autogamous	Anemochory	Ornitophily	Atlantic Forest (SC, MS)	LC	VU	Aguiar-Melo *et al.*, 2020	56
Vriesea gigantea Gaudich.	7	80	11	-	0.620	0.653	-	-	Mixed	Anemochory	Ornitophily, Entomophily, Chiropterophily	Atlantic Forest (SC)	LC	VU	Palma-Silva *et al.*, 2007	3

Subfamily/ Taxa	N pops	N ind	N SSRs	RA	*Ho*	*He*	F_{IS}	F_{ST}	Mating System	Seed Dispersal	Pollination	Biome	NC	LC	Reference	Number
Vriesea gigantea Gaudich	13	431	10	2.82	0.430	0.579	0.273	0.230	Mixed	Anemochory	Ornitophily, Entomophily, Chiropterophily	Atlantic Forest (SP, MG, PR, SC, RS)	LC	VU	Palma-Silva *et al.*, 2009	7
Vriesea gigantea Gaudich	3	26	8	-	-	-	0.428	-	Mixed	Anemochory	Ornitophily, Entomophily, Chiropterophily	Atlantic Forest (RS)	LC	VU	Paggi *et al.*, 2015	25
Vriesea gigantea Gaudich	3	3	4	-	-	-	0.250	0.470	Mixed	Anemochory	Ornitophily, Entomophily, Chiropterophily	Atlantic Forest (RS)	LC	VU	Paggi *et al.*, 2022	71
Vriesea incurvata Gaudich.	-	20	3	-	0.837	0.871	0.021	-	Mixed with Clonal Reproduction	Anemochory	Ornitophily	Atlantic Forest (SC)	LC	VU	Matos *et al.*, 2016	30
Vriesea incurvata Gaudich.	6	29	14	4.67	0.458	0.644	0.292	-	Mixed with Clonal Reproduction	Anemochory	Ornitophily	Atlantic Forest (RS, SC, PR)	LC	VU	Zanella *et al.*, 2016	32
Vriesea incurvata Gaudich.	6	140	13	2.462	0.472	0.631	0.258	0.051	Mixed with Clonal Reproduction	Anemochory	Ornitophily	Atlantic Forest (RS, SC, PR, SP)	LC	VU	Aguiar-Melo *et al.*, 2019	46
Vriesea incurvata Gaudich.	2	35	12	3.311	0.508	0.762	0.369	-	Mixed with Clonal Reproduction	Anemochory	Ornitophily	Atlantic Forest (SC, SP)	LC	VU	Aguiar-Melo *et al.*, 2020	56
Vriesea inflata Wawra (Wawra)	2	11	12	2.37	0.499	0.635	0.250	-	-	-	Ornitophily	Atlantic Forest (SP)	-	DD	Aguiar-Melo *et al.*, 2020	56
Vriesea minarum L.B.Sm.	1	20	10	-	0.432	0.579	0.162	-	Mixed with Clonal Reproduction	Anemochory	Ornitophily, Chiropterophily	Atlantic Forest (MG)	VU	EX	Lavor *et al.*, 2013	17
Vriesea minarum L.B.Sm.	12	206	10	2.552	0.403	0.569	0.341	0.088	Mixed with Clonal Reproduction	Anemochory	Ornitophily, Chiropterophily	Atlantic Forest (SC)	VU	VU	Lavor *et al.*, 2014	21
Vriesea oligantha (Baker) Mez	2	34	19	4.701	0.237	0.557	0.512	-	-	Anemochory	Entomophily, Chiropterophily	(MG, BA)	-	VU	Cacossi *et al.*, 2019	48
Vriesea oligantha (Baker) Mez	12	229	9	3.29	0.294	0.439	0.398	0.432	-	Anemochory	Entomophily, Chiropterophily	(MG, BA)	-	VU	Dantas-Queiroz *et al.*, 2021	68
Vriesea reitzii Leme & And.Costa	6	187	7	4.721	0.436	0.534	0.168	0.0796	Mixed with Clonal Reproduction	Anemochory	Ornitophily	Atlantic Forest (RS, SC, PR)	NT	VU	Soares *et al.*, 2018	43
Vriesea scalaris E.Morren	2	30	15	3.84	0.146	0.323	0.520	-	Mixed	Anemochory	Ornitophily	Atlantic Forest (SC, BA)	LC	EN	Neri *et al.*, 2017 I	36
Vriesea scalaris E.Morren	12	201	15	2.263	0.148	0.363	0.521	0.396	Mixed	Anemochory	Ornitophily	Atlantic Forest (PE,	LC	EN	Neri *et al.*, 2021	69

Subfamily/ Taxa	N pops	N ind	N SSRs	RA	*Ho*	*He*	F_{IS}	F_{ST}	Mating System	Seed Dispersal	Pollination	Biome	NC	LC	Reference	Number
												BA, ES, RJ, SP, SC)				
Vriesea simplex (Vell.) Beer	2	43	10	5.15	0.209	0.456	0.605	-	Mixed	Anemochory	Ornitophily	Atlantic Forest (SP, BA)	EN	NA	Neri *et al.*, 2015	27
Vriesea simplex (Vell.) Beer	2	44	15	7.49	0.550	0.684	0.167	-	Mixed	Anemochory	Ornitophily	Atlantic Forest (RJ)	EN	NA	Neri *et al.*, 2017	36
Vriesea simplex (Vell.) Beer	2	26	12	4.651	0.535	0.680	0.215	-	Mixed	Anemochory	Ornitophily	Atlantic Forest (SP)	EN	NA	Aguiar-Melo *et al.*, 2020	56
Vriesea simplex (Vell.) Beer	9	195	15	4.317	0.548	0.693	0.188	0.098	Mixed	Anemochory	Ornitophily	Atlantic Forest (RJ, SP, ES)	EN	NA	Neri *et al.*, 2021	69
Vriesea taritubensis var. *patens* Neves & A.F. Costa	1	17	12	6.448	0.657	0.824	0.209	-	-	Anemochory	Ornitophily	Atlantic Forest (SP)	-	VU	Aguiar-Melo *et al.*, 2020	56
Werauhia tonduziana (L.B.Sm.) J.R.Grant	14	274	8	-	0.372	0.576	0.293	0.13	Autogamous	Anemochory	Entomophily, Chiropterophily	American Continent (CR)	-	-	Amici *et al.*, 2019	47
**Puyoideae**																
Puya hamata L.B. Sm.	7	74	5	2.16	0.34	0.361	0.088	-	-	Anemochory	Ornitophily	American Continent (EC, CO)	-	-	Rivadeneira *et al.*, 2020	66
Puya hutchisonii L.B.Sm.	1	2	6	-	1.000	0.666	-	-	-	-	-	American Continent (PE)	-	-	Tumi *et al.*, 2019	54
Puya macrura Mez	1	5	13	-	0.504	0.650	-	-	-	-	-	American Continent (PE)	-	-	Tumi *et al.*, 2019	54
Puya macropoda L.B.Sm.	1	4	15	-	0.455	0.559	-	-	-	-	-	American Continent (PE)	-	-	Tumi *et al.*, 2019	54
Puya raimondii Harms	4	64	12	-	0.158	0.201	-	0.209	Mixed	Anemochory	Ornitophily	American Continent (PE)	EN		Tumi *et al.*, 2019	54
Puya raimondii Harms	3	84	12	-	0.050	0.216	0.776	0.426	Mixed	Anemochory	Ornitophily	American Continent (PE)	EN	-	Tumi *et al.*, 2022	72
