# Supplementary material for: Genetic diversity and conservation in Bromeliaceae based on SSR markers
Source: Genet Mol Biol. 2024 Apr 26;46(3 Suppl 1):e20230135. doi: 10.1590/1678-4685-GMB-2023-0135 (PMC11113272; doi:10.1590/1678-4685-GMB-2023-0135)
Supplement: Figure S1 - [file 1415-4757-GMB-46-03-s1-e20230135-s4.pdf]

Genetics and Molecular Biology

https://doi.org/10.1590/1678-4685-GMB-2023-0135

Supplementary Material to "Genetic diversity and conservation in Bromeliaceae based on SSR markers"

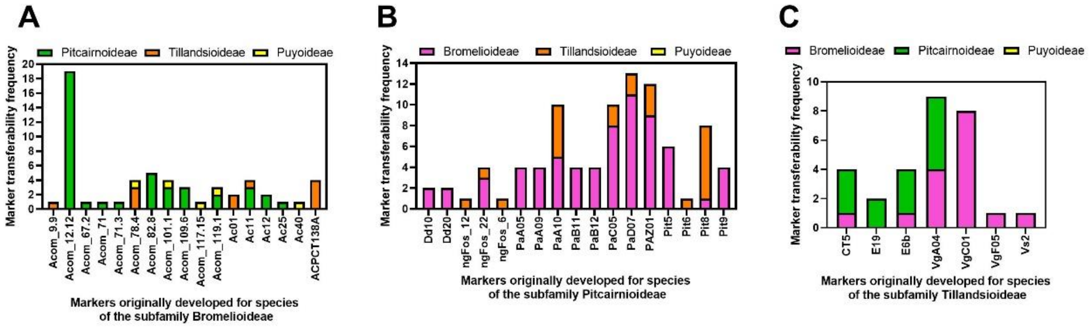

Figure S1 – Microsatellite markers originally developed for species of subfamilies Bromelioideae, Pitcairnoideae and Tillandsioideae used in species of other subfamilies and the frequency of transferability of these markers.
