# Supplementary material for: Genetic diversity and conservation in Bromeliaceae based on SSR markers
Source: Genet Mol Biol. 2024 Apr 26;46(3 Suppl 1):e20230135. doi: 10.1590/1678-4685-GMB-2023-0135 (PMC11113272; doi:10.1590/1678-4685-GMB-2023-0135)
Supplement: Figure S2 - [file 1415-4757-GMB-46-03-s1-e20230135-s5.pdf]

Genetics and Molecular Biology

https://doi.org/10.1590/1678-4685-GMB-2023-0135

Supplementary Material to "Genetic diversity and conservation in Bromeliaceae based on SSR markers"

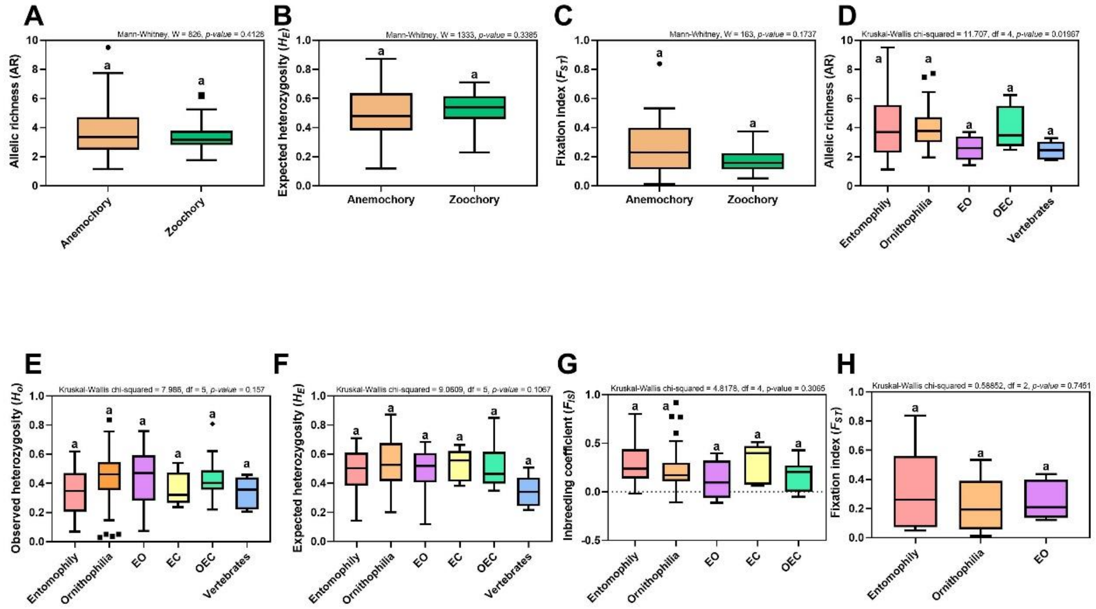

Figure S2 – Box and whisker plots showing the comparisons that were not statistically significant in this study considering a $p \leq 0.05$. Mann-Whitney test was used to seed dispersal mechanisms comparisons: a) Allelic Richness (AR). b) H_E. c) F_{ST}. Kruskal Wallis test with Dunn's post hoc test was applied for pollinator comparisons. d) AR. e) *H*o. f) H_E. g) F_{IS}. h) F_{ST}. Boxes show the 25th, 50th and 75th percentiles; whiskers depict the minimum and maximum values of each variable. EO: Entomophily and Ornithophily; EOC: Entomophily, Ornithophily and chiropterophily; EQ: Entomophily and chiropterophily.
