# Supplementary material for: Genetic diversity and conservation in Bromeliaceae based on SSR markers
Source: Genet Mol Biol. 2024 Apr 26;46(3 Suppl 1):e20230135. doi: 10.1590/1678-4685-GMB-2023-0135 (PMC11113272; doi:10.1590/1678-4685-GMB-2023-0135)
Supplement: Figure S3 - [file 1415-4757-GMB-46-03-s1-e20230135-s6.pdf]

Genetics and Molecular Biology

https://doi.org/10.1590/1678-4685-GMB-2023-0135

Supplementary Material to "Genetic diversity and conservation in Bromeliaceae based on SSR markers"

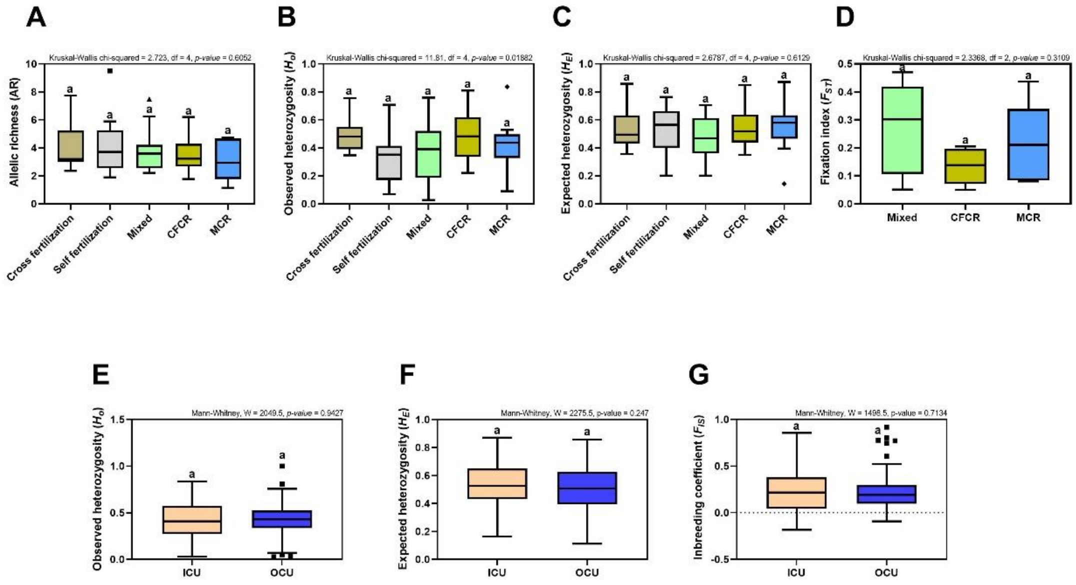

Figure S3 – Box and whisker plots showing the comparisons that were not statistically significant in this study considering a $P \leq 0.05$. Kruskal Wallis test with Dunn's post hoc test was applied for reproductive system comparisons. a) Allelic Richness (AR). b) Ho. c) H_E. d) F_{ST}. Mann-Whitney test was used for the comparisons of taxa sampled inside and outside the conservation unit. e) Ho. f) H_E. g) F_{IS}. Boxes show the 25th, 50th and 75th percentiles; whiskers depict the minimum and maximum values of each variable. ICU: inside conservation unit; OCU: outside conservation unit.
