# Supplementary material for: Genetic diversity and conservation in Bromeliaceae based on SSR markers
Source: Genet Mol Biol. 2024 Apr 26;46(3 Suppl 1):e20230135. doi: 10.1590/1678-4685-GMB-2023-0135 (PMC11113272; doi:10.1590/1678-4685-GMB-2023-0135)
Supplement: Figure S4 - [file 1415-4757-GMB-46-03-s1-e20230135-s7.pdf]

*Genetics and Molecular Biology*

https://doi.org/10.1590/1678-4685-GMB-2023-0135

# Supplementary Material to "Genetic diversity and conservation in Bromeliaceae based on SSR markers"

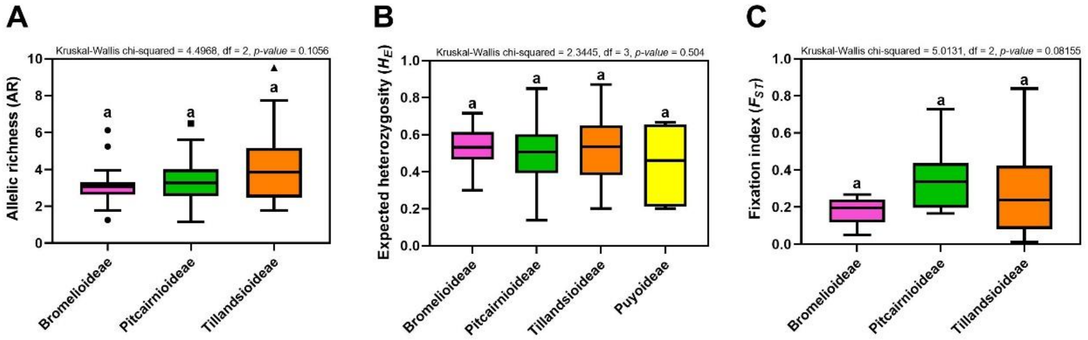


**Figure S4** – Box and whisker plots showing the comparisons that were not statistically significant in this study considering a $p \leq 0.05$. Kruskal Wallis test with Dunn's post hoc test was applied for subfamily comparisons. a) Allelic Richness (AR). b) $H_E$. c) $F_{ST}$. Boxes show the 25th, 50th and 75th percentiles; whiskers depict the minimum and maximum values of each variable.
