# Supplementary material for: Genetic diversity and conservation in Bromeliaceae based on SSR markers
Source: Genet Mol Biol. 2024 Apr 26;46(3 Suppl 1):e20230135. doi: 10.1590/1678-4685-GMB-2023-0135 (PMC11113272; doi:10.1590/1678-4685-GMB-2023-0135)
Supplement: Figure S5 - [file 1415-4757-GMB-46-03-s1-e20230135-s8.pdf]

*Genetics and Molecular Biology*

https://doi.org/10.1590/1678-4685-GMB-2023-0135

# Supplementary Material to "Genetic diversity and conservation in Bromeliaceae based on SSR markers"

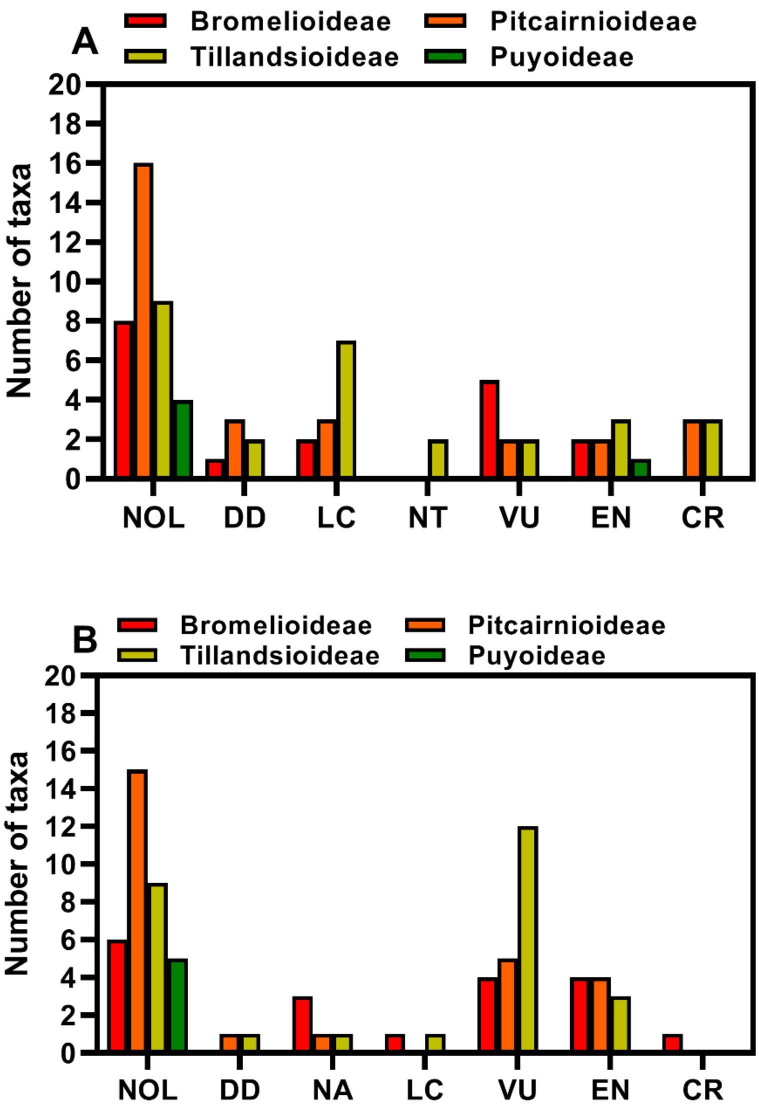


**Figure S5** – Conservation status of the taxa distributed in the four subfamilies retrieved in this study and their threat category. a) National list. b) Subnational list. NOL: not listed in any threat category; DD: Data Deficient; NA: Not threatened; LC: Least Concern; NT: Near Threatened; VU: Vulnerable; EN: Endangered; CR: Critically Endangered.
